# LB-100 Enhances Drugs Efficacy Through Inhibition of P-Glycoprotein Expression in Multidrug-Resistant Glioblastoma and Non-Small Cell Lung Carcinoma Cellular Models

**DOI:** 10.3390/pharmaceutics17020189

**Published:** 2025-02-04

**Authors:** Ana Podolski-Renić, Margarita Chigriai, Sofija Jovanović Stojanov, Marija Grozdanić, Ema Lupšić, Igor Nikolić, Miodrag Dragoj, Jelena Dinić, Milica Pešić

**Affiliations:** 1 Institute for Biological Research “Siniša Stanković”—National Institute of the Republic of Serbia, University of Belgrade, Despota Stefana 142, 11108 Belgrade, Serbia; ana.podolski@ibiss.bg.ac.rs (A.P.-R.); sofija.jovanovic@ibiss.bg.ac.rs (S.J.S.); marija.grozdanic@ibiss.bg.ac.rs (M.G.); ema.lupsic@ibiss.bg.ac.rs (E.L.); miodrag.dragoj@ibiss.bg.ac.rs (M.D.); jelena.dinic@ibiss.bg.ac.rs (J.D.); 2 Advitam Laboratory, Mihaila Suskalovica 13, 11030 Belgrade, Serbia; chigriai@advitamlab.com; 3 Clinic for Neurosurgery, Clinical Center of Serbia, Pasterova 2, 11000 Belgrade, Serbia; igor.nikolic@med.bg.ac.rs; 4 School of Medicine, University of Belgrade, Doktora Subotića 8, 11000 Belgrade, Serbia

**Keywords:** glioblastoma, non-small cell lung carcinoma, multidrug resistance, P-glycoprotein, protein phosphatase 2A, LB-100, doxorubicin, adavosertib

## Abstract

**Background/Objectives**: This study explores the potential of LB-100 (a protein phosphatase 2A—PP2A inhibitor) combined with adavosertib (a WEE1 kinase inhibitor) and doxorubicin (DOX), to overcome multidrug resistance (MDR) in cancer cells and enhance treatment efficacy. **Methods**: We evaluated LB-100 combinations with adavosertib and DOX in patient-derived glioblastoma and non-small cell lung carcinoma cells (NSCLCs) using a real-time cell analyzer. Effectiveness was also assessed through immunofluorescence assay, and interactions were analyzed via SynergyFinder+. We also examined P-glycoprotein (P-gp) expression and drug resistance genes’ expression in MDR glioblastoma and NSCLCs after LB-100 treatment, as well as LB-100 sensitizing effect on DOX and DOX accumulation. **Results**: LB-100 significantly boosts the effectiveness of adavosertib and DOX after multiple applications. It also enhances these drugs’ cytotoxicity in a single application without acting synergistically. Additionally, LB-100 reduces P-gp expression in MDR glioblastoma and NSCLCs, sensitizing them to DOX and increasing its accumulation. **Conclusions**: LB-100 enhances the effectiveness of drugs against MDR cancer cells, presenting a promising strategy to overcome drug resistance in glioblastoma and NSCLCs through P-gp modulation.

## 1. Introduction

Cancer remains one of the leading causes of death worldwide and requires continuous improvement in treatment strategies [1]. A hallmark of cancer is aberrant oncogenic signaling triggered by both genetic and non-genetic alterations [2]. Novel targeted therapies that selectively inhibit activated oncogenic signaling pathways have led to more effective treatments and better outcomes [3]. Unfortunately, these targeted therapies in advanced cancers often do not result in a long-lasting response due to the emergence of resistance. Secondary mutations that lead to reactivation of the oncogenic pathway in the presence of the drug result in resistance to targeted therapies [4,5]. This underscores the need for alternative approaches to effectively combat cancer that are fundamentally different from inhibiting oncogenic signaling.

Recent studies demonstrate that hyperactivation of oncogenic signaling pathways can be as lethal to cancer cells as inhibition of these pathways [6,7]. Indeed, oncogenic signaling in cancer cells is associated with increased activation of stress response pathways that enable cells to cope with the stress caused by oncogenic activity [8]. As a result, further activation of mitogenic signaling could destabilize the fragile homeostasis of cancer cells and overload the stress response pathways [9].

Protein phosphatase 2A (PP2A) is a serine/threonine phosphatase that plays an important role in the progression of mitosis and the cellular response to DNA damage [10]. Due to its ability to control various oncogenic signaling pathways, PP2A has been recognized as a tumor suppressor, leading to the development of drugs designed to reactivate its activity [11]. However, inhibition of PP2A has gained interest as a strategy to target tumors that are resistant to conventional treatments [12]. Namely, the proper function of PP2A during the G2 phase of the cell cycle is essential for cell senescence [13]. Cancer cell senescence is a major contributor to resistance to therapy, especially to conventional DNA-damaging agents that preferentially affect actively dividing cells [14,15]. Recent studies have shown that inhibition of PP2A can help drive senescent cancer cells into mitosis, leading to cell death by mitotic catastrophe [16,17].

LB-100, a small molecule inhibitor of PP2A, induces mitotic catastrophe in several tumor models, especially in combination with DNA-damaging agents [18]. A recent study has shown that LB-100 enhances oncogenic signaling and activates stress response pathways [16]. Lu et al. discovered that LB100 caused an inappropriate entry into mitosis and increased cell death when combined with doxorubicin in glioblastoma cells [19]. In vivo, the combination therapy showed a better response, exhibiting either growth inhibition or regression, than each agent used alone. In addition, the combination of LB-100 with doxorubicin effectively prevented tumor recurrence [19]. It has also been shown that simultaneous inhibition of PP2A by LB-100 and WEE1 kinase by adavosertib is highly lethal in several cancer models, leading to DNA replication breakdown and triggering premature mitosis with subsequent cell death [16]. Importantly, in a phase I clinical trial, LB-100 demonstrated a favorable toxicity profile at doses associated with clinical response [20]. These properties of LB-100 make it an attractive candidate to test the concept of activating oncogenic signaling for cancer treatment.

LB-100 is an emerging pharmaceutical agent with significant potential for overcoming multidrug resistance (MDR) in cancer treatment. Its unique mechanism enables the sensitization of cancer cells to conventional therapies, including radio and chemotherapy [21]. Research has demonstrated that LB-100 can effectively reverse resistance to cisplatin in models of ovarian carcinoma and medulloblastoma [20]. Furthermore, its efficacy in combination with immunotherapy underscores its potential in diverse treatment strategies [22]. The combination approaches with LB-100 target both primary tumors and address emerging resistance mechanisms. Due to its efficacy and safety profile in combination therapies [21], LB-100 is a promising option for combating MDR in cancer.

Glioblastoma (GBM) is the most common primary brain tumor and accounts for about 80% of all tumors of the central nervous system [23]. GBM represents a major challenge in the field of oncology due to its aggressive nature and resistance to conventional therapies [24]. In addition, the low biodistribution of most cancer therapeutics in the brain due to the high expression of P-glycoprotein (P-gp) in the blood–brain barrier (BBB) is one of the limitations in GBM treatment. P-gp or the ATP-binding cassette (ABC) transporter (ABCB1) prevents cellular uptake of a variety of anticancer drugs, leading to cancer drug resistance [25]. Standard treatment options for GBM include maximal surgical resection followed by radiotherapy with concomitant and adjuvant temozolomide administration [26]. Although the standard treatment for GBM is associated with poor response rates, it has remained essentially unchanged over the past decades due to the lack of therapeutic alternatives [27]. As no therapy shows a promising response, there is an urgent need for innovative therapeutic approaches for GBM.

Using glioblastoma cells, patient-derived cells and pairs of sensitive and MDR cancer cell lines, we investigated the anticancer effect of LB-100 in combination with adavosertib, an inhibitor of WEE1 kinase, and doxorubicin, an inhibitor of topoisomerase II. These drugs were selected for their ability to target stress response pathways, particularly DNA damage stress, by either inducing DNA damage stress (doxorubicin) or inhibiting the response to DNA damage (adavosertib). The combined treatment of LB-100 with adavosertib or doxorubicin was additionally investigated in non-small cell lung carcinoma cells. For the first time, we report that LB-100 synergizes with doxorubicin in simultaneous and subsequent treatment by inhibiting P-gp expression.

## 2. Materials and Methods

### 2.1. Compounds

LB-100 and adavosertib were purchased from Selleckchem, Houston, TX, USA. Doxorubicin (DOX) was obtained from Sigma–Aldrich Chemie GmbH, Hamburg, Germany. LB-100 was diluted in sterile deionized water and kept as 10 mM aliquots at −20 °C. Adavosertib and DOX were dissolved as 10 mM stocks in dimethyl sulfoxide (DMSO) and kept as aliquots at −20 °C. Prior to administration, the compounds were dissolved in sterile deionized water.

### 2.2. Chemicals and Reagents

The following chemicals and reagents were used in the experimental work: minimum essential medium (MEM), Ham’s F12 growth media, Dulbecco’s modified minimal essential medium (DMEM), L-glutamine, hydrocortisone, insulin, adenine, DMSO, 3-[4,5-dimethylthiazol-2-yl]-2,5-diphenyltetrazolium bromide (MTT), Hoechst 33342 (Sigma–Aldrich Chemie GmbH, Taufkirchen, Germany), a mixture of antibiotics penicillin-streptomycin, a mixture of antibiotics and antimycotics: penicillin, streptomycin, amphotericin B, MEM non-essential amino acids (Capricorn Scientific GmbH, Ebsdorfergrund, Germany), trypsin/EDTA (Gibco, Thermo Fisher Scientific, Waltham, MA, USA), RPMI 1640 medium, fetal bovine serum (FBS) (Corning, NY, USA), propidium iodide (PI) (Molecular Probes, Thermo Fisher Scientific, USA), ribonuclease A (Invitrogen, Thermo Fisher Scientific, USA), Anti-phospho-histone H2A.X (Ser139) rabbit (Cell Signaling Technology^®^, USA), Tumor Dissociation Kit (Miltenyi Biotec, Bergisch Gladbach, Germany), epidermal growth factor (BioLegend, San Diego, CA, USA), anti-cytokeratin 8/18 (CK8/18) primary antibody cocktail (clone SU0338, #MA5-32118), anti-ABCB1 monoclonal antibody (clone C219, #MA1-26528), isotype control IgG2bk (Abcam, Cambridge, UK), Alexa Fluor 555 goat anti-mouse secondary antibody (#A-21422), Alexa Fluor 488 goat anti-rabbit secondary antibody (#A-11008) (Thermo Fisher Scientific, Waltham, MA, USA), polyclonal rabbit anti-glial fibrillary acidic protein antibody (anti-GFAP, Z0334) (Agilent Dako, Santa Clara, CA, USA) and PE-conjugated anti-P-gp antibody (BD Biosciences, Plymouth, UK).

### 2.3. Cells and Cell Culture

Glioblastoma cell line U87 and human non-small cell lung carcinoma cells (NSCLCs) NCI-H460 were purchased from the American Type Culture Collection (Rockville, MD, USA). Multidrug-resistant (MDR) U87-TxR cell line was selected from U87 by continuous exposure to the stepwise increasing concentrations of paclitaxel during nine months [28], while MDR NCI-H460/R cells were selected from NCI-H460 cells by continuous exposure to the stepwise increasing concentrations of DOX during three months [29]. Both MDR models are well-characterized and detailed in several publications [30,31,32]. A key characteristic contributing to the MDR phenotype is the overexpression of P-glycoprotein (P-gp). U87 and U87-TxR were cultivated in MEM, supplemented with 10% FBS, 2 mM L-glutamine, 5000 U/mL penicillin and 5 mg/mL streptomycin mixture, and 1% non-essential amino acids. NCI-H460 and NCI-H460/R were grown in RPMI 1640 medium, supplemented with 10% FBS, 2 mM L-glutamine, and 10,000 U/mL penicillin, 10 mg/mL streptomycin, 25 mg/mL amphotericin B solutions. All cell lines were grown at 37 °C in a humid atmosphere with 5% CO_2_. The cells were grown in 25 cm^2^ flasks (Sarstedt, Nümbrecht, Germany) until they reached 80–90% confluence. Then, cell passage was carried out using 0.25% trypsin/EDTA. The cells were counted using a Bürker–Türk hemocytometer on an inverted microscope. Once counted, the cells were seeded at the following densities for further experimentation or maintenance in culture: 8000 cells/ cm^2^ for NCI-H460 and NCI-H460/R, and 16,000 cells/cm^2^ for U87 and U87-TxR.

### 2.4. Patient-Derived Cell Cultures

A sample DT7 from patient with WHO grade 4, IDH-wildtype glioblastoma was collected from the Clinic for Neurosurgery at the Clinical Center of Serbia after obtaining the patient’s informed consent and the approval from the Ethics Committee of the Clinical Center of Serbia (ref. number 187/13). The histological grade was determined by correlating neuroradiological examination and histopathological analysis of the surgical specimen. The expression of GFAP, OLIG2, MAP2, vimentin and p53 were confirmed by histopathological analysis. TR159, an NSCLC sample, was obtained from the Clinic for Thoracic Surgery at the Clinical Center of Serbia after obtaining informed consent from the patients and approval from the Ethics Committee of the Clinical Center of Serbia (ref. number 623/4). The sample was collected during surgery, and histopathologic analysis determined NSCLC diagnosis, histologic grade, stage, necrosis and lymph node invasion status. The histological stage of the collected NSCLC sample is IIA. The surgical specimens DT7 and TR159 were placed in a sterile tube containing antibiotic-antimycotic solution and immediately transported to the research laboratory for further processing.

To establish primary cultures, the tissue was manually minced using a surgical blade in a sterile Petri dish immediately upon arrival at the laboratory. The samples were then sectioned into pieces measuring 3–5 mm and dissociated with the Tumor Dissociation Kit (Miltenyi Biotec, Bergisch Gladbach, Germany) as per the manufacturer’s protocol. The tissue was incubated on a 37 °C orbital shaker (KS 4000 ic control, IKA, Königswinter, Germany) at 300 rpm for 90 min. Following incubation, the dissociated material was transferred to DMEM/Ham’s F12 medium (1:3 ratio) enriched with 5% FBS, antibiotic-antimycotic solution, 4 μg/mL hydrocortisone, 1 μg/mL insulin, 10 ng/mL epidermal growth factor and 24 μg/mL adenine. The tissue was cultured in 25 cm^2^ cell culture flasks, with the medium replaced after cell attachment was observed. Successfully established patient-derived cultures were maintained in a 37 °C humidified atmosphere with 5% CO_2_ and grown to confluence before experiments.

### 2.5. Real-Time Primary Culture Cells Growth Monitoring

Growth of DT7 and TR159 primary culture cells was monitored in real time using the xCELLigence system (Roche, Basel, Switzerland) with a 96-well E-plate. To establish a baseline, 100 µL of medium was added to each well, and the background was measured before seeding the cells. The cells were then seeded, and the total volume in each well was adjusted to 200 µL with media. Twenty-four hours after seeding, cells were treated with 250 and 500 nM DOX and adavosertib, either individually or in combination with 2.5 µM and 5.0 µM LB-100. Drug treatments were refreshed every 2 to 3 days for a total of 10 days. After the treatment period, the TR159 cells were allowed to recover for another 5 days. The xCELLigence system automatically recorded impedance values for each well every 30 min and displayed the results as a cell index (CI). Antiproliferative effects are characterized by a reduced growth rate while maintaining a similar growth trajectory to untreated cells. Cytostatic effects are identified by a growth profile that remains consistent with the initial seeding baseline, whereas cytotoxic effects refer to a decline in the growth profile below the baseline established during seeding.

### 2.6. Immunofluorescence Assay

The immunofluorescence assay was optimized to distinguish cancer cells from stromal cells using GFAP antibody and CK8/18 antibody mixture cocktail in glioblastoma and NSCLC primary cell cultures, respectively. In the glioblastoma primary cell culture, GFAP-positive cells were considered as glioblastoma cells. In the NSCLC primary cell culture, CK8/18-positive cells were considered as cancer cells. The immunofluorescence assay was additionally optimized to quantify P-gp expression using ABCB1 antibody. Patient-derived cells were seeded in black, clear-bottom 384-well cell culture microplates (Thermo Fisher Scientific, Waltham, MA, USA) in 50 µL of cell growth medium at a density of 4000 cells per well for glioblastoma primary cells and 1000 cells per well for NSCLC primary cells. The glioblastoma primary cells were treated 24 h after seeding with 250, 500, 1000 and 2500 nM adavosertib and DOX alone or in combination with 0.5, 1.0 and 2.5 µM LB-100 for 5 days. NSCLC primary cells were treated with the same concentrations of adavosertib and DOX alone or in combinations with 250, 500 and 1000 nM LB-100 for 7 days. After the treatments, the cells were fixed in 4% paraformaldehyde for 20 min at RT and washed using the Wellwash™ Versa microplate washer (Thermo Fisher Scientific, Waltham, MA, USA). The cells were then blocked with 2% bovine serum albumin (BSA) in PBS for 1 h at RT. After blocking, the glioblastoma and NSCLC primary cells were incubated overnight at 4 °C with a primary rabbit GFAP antibody and primary rabbit CK8/18 antibody cocktail, respectively. Both primary culture cells were co-immunostained with mouse antibody against ABCB1 to detect the presence of P-gp in both the cancer and stromal cells. The cells were washed three times with PBS using the microplate washer before incubation with the secondary Alexa Fluor 555 goat anti-mouse antibody and the secondary Alexa Fluor 488 goat anti-rabbit antibody at RT for 2 h in the dark. The cell nuclei were counterstained with 1 µg/mL Hoechst 33342 at RT for 2 h. The cells were kept at 4 °C in the dark until imaging. Fluorescently labeled cells were visualized using the ImageXpress Pico Automated Cell Imaging System (Molecular Devices, San Jose, CA, USA) with a 4× objective lens, after determining optimal exposure times for each illumination filter (Figure S1). Image analysis was conducted using CellReporterXpress software v. 2.8.2.669 (Molecular Devices). Cytotoxicity of the compounds was assessed using the Cell Scoring Analysis Protocol, as previously described [33]. The Multi-Wavelength Cell Scoring Analysis Protocol was used to evaluate ABCB1 expression [33].

### 2.7. Drug Interaction Assessment

SynergyFinder+ is an advanced online tool used to analyze interactions between drugs, specifically LB-100 combined with adavosertib and LB-100 combined with DOX, to identify potential synergies. We collected dose–response data from immunofluorescence assays performed on DT7 and TR159 patient-derived cells for various drug combinations. These data were organized into a matrix, and SynergyFinder+ utilized several models to evaluate the synergies between the drugs. The Zero Interaction Potency (ZIP) model enhances the analysis by comparing the drugs’ dose–response curves. It assumes that there is no interaction between the drugs and calculates the expected effect accordingly. Any deviations from this expected effect are interpreted as a measure of synergy [34]. Next, the Bliss Independence model assesses the scenario where the drugs act independently. It estimates the expected effect based on the probability of unrelated events occurring simultaneously. If the observed effects of the combination exceed this expected value, it indicates a synergistic interaction [34]. Additionally, the Loewe Additivity model is based on the principle that drugs in combination behave like a single drug administered at varying doses. This model helps to determine whether the combined doses result in an additive effect or provide evidence of synergy [34]. Finally, the Highest Single Agent (HSA) model compares the effects of the drug combination to the maximum effects of the individual drugs. If the combination’s effect surpasses that of the most effective single agent, it suggests a synergistic interaction [34]. For all the four models, observing synergism is indicated by a positive value above zero.

### 2.8. MTT Assay

The combined effects of LB-100 with DOX were studied in MDR U87-TxR glioblastoma and NCI-H460/R NSCLC cell lines by the MTT assay. The assay works by measuring the metabolic activity of the cell, which indirectly measures the viability of living cells. The cells were seeded in 96-well microtiter plates at the density of 2000 cells per well for NCI-H460/R and 4000 cells per well for U87-TxR in 100 μL of the appropriate medium. In simultaneous treatments that lasted 72 h, two concentrations of LB-100 (2.5 and 5 µM) were combined with DOX (100, 250, 500, 1000 and 2500 nM) in MDR glioblastoma cells, as well as with DOX (50, 100, 250, 500 and 1000 nM) in MDR NSCLCs. In subsequent treatment, both MDR cancer cell lines were pre-treated with 2.5 µM and 5 µM LB-100. After 24 h, the cells were treated with increasing concentrations of DOX in the same concentrations range like in simultaneous treatments. The experiment was carried out for further 48 h. At the end of the treatment period, MTT was introduced to each well at a final concentration of 0.2 mg/mL, and the plates were stored for 3 h at 37 °C in a 5% CO_2_ environment. The formazan crystals formed in the cells with functioning mitochondria were dissolved in 100 µL of DMSO, and absorbance readings were taken at 570 nm with a reference wavelength of 690 nm using the Multiskan Sky microplate reader (Thermo Scientific, Waltham, MA, USA). The IC50 value, representing the drug concentration required to inhibit cell growth by 50%, was calculated through non-linear regression analysis using the log (inhibitor) vs. normalized response model in GraphPad Prism 8.0.2 software.

### 2.9. Flow Cytometric Detection of P-gp Expression

The P-gp expression levels in U87-TxR and NCI-H460/R cells were measured by flow cytometry. The cells were seeded in adherent 6-well plates, incubated overnight and treated with 2.5 µM and 5.0 µM LB-100. After 24 h, 48 h and 72 h, cells were collected by trypsinization, washed in PBS and directly immunostained by PE-conjugated anti-P-gp antibody according to the manufacturer’s protocol. In all experiments, unstained untreated sensitive (U87 and NCI-H460) cancer cells were utilized as negative controls. Each experiment also incorporated an isotype control IgG2bk to assess the background fluorescence levels. The fluorescence intensity was measured on a CytoFLEX flow cytometer (Beckman Coulter, Indianapolis, IN, USA) in the red (585 nm) channel. Each sample recorded at least 10,000 events, and the obtained results were analyzed using CytExpert 2.4 software (Beckman Coulter, Indianapolis, IN, USA).

### 2.10. qRT-PCR

Quantitative real-time PCR (qPCR) was used to determine the mRNA expression level of *MDR1* (*ABCB1*) [35], *HIF-1α* [36], *MGMT* [37], *PARP1* and *PARP2* in U87-TxR and NCI-H460/R cells. The primer sets used to amplify *PARP1* and *PARP2* were generously provided by the Department of Molecular Biology at the Institute for Biological Research “Siniša Stanković”—National Institute of the Republic of Serbia, University of Belgrade. *PARP1:* forward 5′-GTGGATCCTGATTCTGGACTGG-3′ and reverse 5′-TCCTTGGACGGCATCTGTTC-3′ and *PARP2*: forward 5′-CTACACCAGGATTCCGCATGA-3′ and reverse 5′-GTGTGGGAGCATGGGTAGATT-3′. The reactions were performed using a Maxima SYBR Green/ROX qPCR Master Mix (Thermo Scientific, Waltham, MA, USA) on an ABI PRISM 7000 Sequence Detection System (Applied Biosystems, Waltham, MA, USA) according to the manufacturer’s recommendations. Each sample was tested in triplicate, and relative gene expression levels were analyzed by the 2^−ΔΔCt^ method [38]. The difference between the Ct values of specific genes and the endogenous control (*ACTB*) [39] represents the Δ^Ct^ value. All statistical analyses and data visualizations were performed using the R statistical computing environment (Version 4.2.2; R Core Team). Graphs and figures were generated with the ggplot2 package (Version 3.5.1), and statistical comparisons were conducted using the rstatix package (Version 0.7.2).

### 2.11. Rho123 and DOX Accumulation Assays

The accumulation of fluorescent Rho123 was analyzed by flow cytometry. Since Rho123 is a P-gp substrate, the intensity of fluorescence is proportional to Rho123 accumulation in the cell. NCI-H460/R and U87-TxR cells were counted, suspended in a growth medium and treated with increasing concentrations of LB-100 (2.5, 5, 10, 25 and 50 µM) and Rho123 (2 µM) simultaneously. The samples were then incubated at 37 °C in 5% CO_2_ for 30 min. At the end of the accumulation period, the samples were pelleted by centrifugation, washed with PBS and placed in 1 mL PBS. The green channel fluorescence (525 nm) of the samples was read on a CytoFLEX flow cytometer (Beckman Coulter, Indianapolis, IN, USA), and the results were analyzed by CytExpert software (Beckman Coulter, Indianapolis, IN, USA).

DOX fluorescence was utilized to examine the uptake of DOX in U87-TxR and NCI-H460/R cells after a 72 h treatment with 2.5 and 5.0 µM LB-100, using flow cytometry. DOX is a substrate for P-gp and emits fluorescence, with the intensity indicating the amount of DOX accumulated by the cells. For the analysis, both untreated and LB-100 treated cells were counted, and 100,000 cells per sample were placed in a growth medium containing 10 µM DOX after the 72 h treatment. The samples were incubated at 37 °C with 5% CO_2_ for 60 min. After the accumulation period, the samples that underwent centrifugation were washed twice with cold PBS and then resuspended in 1 mL of PBS for further analysis. The red channel fluorescence at 610 nm was measured using a CytoFLEX flow cytometer (Beckman Coulter, Indianapolis, IN, USA), and the results were analyzed with CytExpert software (Beckman Coulter, Indianapolis, IN, USA).

### 2.12. SwissADME Online Tool

The pharmacokinetic and drug-likeness properties of LB-100 were analyzed using the SwissADME website. Using this platform, values such as WLOGP (water partition coefficient, which indicates the lipophilicity of a molecule) and TPSA (topological polar surface area, which represents the polar surface area of a molecule) were calculated for LB-100 [40]. The resulting “boiled egg” model indicates the position of the molecule on a WLOGP-versus-TPSA diagram and facilitates the assessment of passive gastrointestinal absorption and brain penetration of small molecules such as LB-100. The white region of the diagram corresponds to a high probability of passive absorption through the human intestine (HIA), while the yellow region, the yolk, indicates a high probability of blood–brain barrier (BBB) penetration. These regions are not mutually exclusive. Blue-colored dots indicate molecules that are expected to be actively secreted by P-gp (PGP+), whereas red-colored dots represent molecules that are not considered P-gp substrates (PGP−).

### 2.13. Cell Cycle Analysis

The MDR cancer cells were seeded in 6-well plates at a density of 200,000 cells per well for U87-TxR, and 100,000 cells per well for NCI-H460/R in 2 mL of the appropriate medium. The effects and the aforementioned subsequent treatment of 2.5 μM LB-100 and 250 nM DOX in U87-TxR cells, and 5 μM LB-100 and 500 nM DOX in NCI-H460/R cells on cell cycle distribution were studied after 72 h. Following treatment, cells were trypsinized, pelleted by centrifugation, rinsed with PBS and fixed in 70% ethanol for 24 h at −20 °C. After fixation, the cells were rinsed with PBS and pretreated with 50 μg/mL ribonuclease A, followed by a 15 min incubation at 37 °C. Next, PI was added to a final concentration of 2 μg/mL. Flow cytometry was performed using the CytoFLEX flow cytometer (Beckman Coulter, Indianapolis, IN, USA), collecting data from at least 10,000 events per sample. The cell cycle distribution was analyzed using CytExpert software (Beckman Coulter, Indianapolis, IN, USA).

### 2.14. Double Strand DNA Breaks Detection

To assess DNA damage in U87-TxR and NCI-H460/R cells, the fluorescence intensity of the anti-phospho-histone H2A.X antibody was employed. This antibody identifies endogenous phosphorylated H2A.X, which accumulates at the sites of double-strand breaks in DNA. The cells were initially plated at a concentration of 100,000 cells per well in 6-well tissue culture plates and allowed to grow overnight. The effects of the aforementioned subsequent treatment of 2.5 μM LB-100 and 250 nM DOX in U87-TxR cells, and 5 μM LB-100 and 500 nM DOX in NCI-H460/R cells were assessed. After treatment, cells were harvested, rinsed with PBS and fixed in 4% paraformaldehyde for 15 min at room temperature. Next, they were permeabilized with ice-cold 90% methanol for 90 min at −20 °C. After another PBS rinse, the cells were blocked with 0.5% BSA in PBS for 1 h and incubated overnight at 4 °C with an anti-phospho-histone H2A.X antibody (1:500 dilution in 0.5% BSA in PBS). After another PBS wash, the cells were incubated for 30 min at room temperature with the fluorescent Alexa Fluor 488 anti-rabbit IgG (H + L) secondary antibody (1:1000 dilution in 0.5% BSA in PBS). The cells were washed again and resuspended in 1 mL of PBS, and green fluorescence intensity was measured at 525 nm using a CytoFLEX flow cytometer (Beckman Coulter, Indianapolis, IN, USA). A minimum of 10,000 events were recorded for each sample, and the results were analyzed using CytExpert software (Beckman Coulter, Indianapolis, IN, USA).

### 2.15. Statistical Analysis

The IC50 values obtained from the immunofluorescence and MTT assays were calculated using nonlinear regression analysis in GraphPad Prism 8.0.2 for Windows (San Diego, CA, USA). The results regarding cytotoxicity and the percentage of P-gp positive cells in patient-derived samples, assessed by the immunofluorescence assay, as well as P-gp expression in MDR cancer cell lines, were analyzed using a two-way ANOVA followed by Dunnett’s multiple comparisons test, also using GraphPad Prism 8.0.2. A one-way ANOVA with Dunnett’s multiple comparisons test was applied for the DOX accumulation assay, while the qRT-PCR results were analyzed using a paired *t*-test. *p* values below 0.05 were considered significant (* *p* < 0.05; ** *p* < 0.01; *** *p* < 0.001; n.s. = non-significant). All the results represent the means ± SEM from at least three samples or experiments.

## 3. Results

### 3.1. Assessment of LB-100 Combinations with Adavosetib and Doxorubicin Across Multiple Application Cycles Using a Real-Time Cell Analyzer

**Figure 1:**
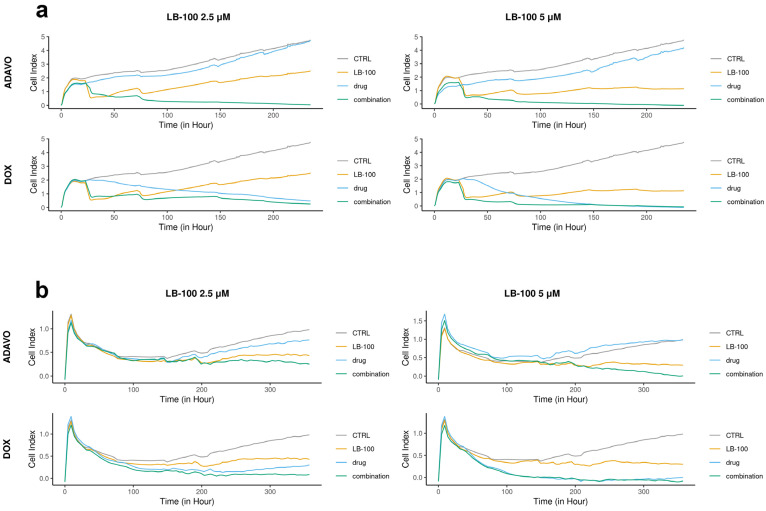
Cell proliferation rates monitored over time using the xCELLigence system. DT7 glioblastoma cells (**a**) were observed for 235 h, while TR159 NSCLCs (**b**) were monitored for 357 h. The results were obtained by treating patient-derived DT7 cells and TR159 cells with LB-100 at concentrations of 2.5 µM and 5.0 µM, as well as adavosertib (ADAVO) and doxorubicin (DOX) at concentrations of 250 nM and 500 nM. The treatments also included combinations of LB-100 with ADAVO and LB-100 with DOX. The Cell Index (CI) of the control cells (on the y-axis) was compared to the CI of the drug-treated cells and their combinations. The results were analyzed using the RTCA software for single plate (SP) analyses.

We assessed the toxicity of LB-100 in combination with compounds, which target stress response pathways, specifically adavosertib and DOX, by applying several treatment cycles on patient-derived cells DT7 and TR159. We analyzed the efficacy of these combinations in inhibiting cell growth in real time using the RTCA xCELLigence system (Figure 1).

In the DT7 cells, we observed an antiproliferative effect at concentrations of 250 nM and 500 nM of adavosertib and 2.5 µM of LB-100 alone. However, a higher concentration of LB-100 (5.0 µM) resulted in a cytostatic effect (Figure 1a). Both combinations of LB-100 and adavosertib exhibited cytotoxic effects, demonstrating a significant synergistic effect. Additionally, including 2.5 µM LB-100 notably enhanced the cytostatic effect of 250 nM DOX (Figure 1a). A synergy was also observed with higher concentrations of LB-100, as adding 5.0 µM LB-100 to 500 nM DOX immediately induced a cytotoxic effect (Figure 1a).

In the case of TR159, both concentrations of adavosertib demonstrated antiproliferative effects, while LB-100 at both concentrations exhibited a cytostatic effect (Figure 1b). The combination of 2.5 µM LB-100 and 250 nM adavosertib enhanced the cytostatic effect. Furthermore, adding 5.0 µM LB-100 to 500 nM adavosertib resulted in a significant cytotoxic effect, indicating a notable synergy between these drugs (Figure 1b). Both combinations of doxorubicin (DOX) with LB-100 showed a similar cytotoxic pattern on cell growth as DOX alone (Figure 1b).

### 3.2. LB-100 Increases the Effectiveness of Adavosertib and Doxorubicin After a Single Application Cycle as Assessed by Immunofluorescence Assay

Table 1 illustrates the effects of combining LB-100 with adavosertib and DOX on patient-derived cells from glioblastoma (DT7) and NSCLCs (TR159). The results are presented as IC50 values in nM.

In DT7 cells, LB-100 alone exhibits a relatively high IC50 value (above 10 µM), indicating lower efficacy compared to other treatments. Adavosertib, when tested alone, has a lower IC50 value of around 5 µM. However, when LB-100 is combined with adavosertib, there is a significant reduction in IC50 values, particularly with concentrations of 1000 nM and 2500 nM of LB-100 (3.5 µM, *p* < 0.05, and 2.4 µM, *p* < 0.01, respectively). This demonstrates that LB-100 enhances the potency of adavosertib in treating glioblastoma. In comparison, DOX shows a considerably lower IC50 value of approximately 600 nM, reflecting its high potency. Furthermore, when combined with LB-100 at 2500 nM, the IC50 value decreases significantly to 400 nM (*p* < 0.001), underscoring LB-100’s ability to enhance the effectiveness of DOX.

In TR159 cells, LB-100 displays an IC50 value that is more than 10-fold lower than that observed in DT7 cells (around 900 nM), indicating that these cancer cells are more sensitive to LB-100. Adavosertib’s performance in this context is similar to that in DT7 cells, with an IC50 value of around 400 nM, which is lower than that of LB-100. The combinations of LB-100 consistently reduce the IC50 values for adavosertib across all settings (*p* < 0.05), demonstrating its ability to enhance the effects of adavosertib. Although DOX has the lowest IC50 value of all treatments in TR159 at 90 nM, the combination with LB-100 results in negligible reductions in IC50 values that lack statistical significance.

### 3.3. Nature of Interaction Between LB-100 and Adavosertib/Doxorubicin Assessed by SynergyFinder+

Table 2 and Figures S2–S5 summarize the interactions between LB-100 combined with adavosertib and DOX in DT7 and TR159 cells. Zero Interaction Potency (ZIP) is a model that assumes drugs function independently without interacting with one another. When applying this model to the combination of LB-100 and adavosertib, slightly negative scores in DT7 (−0.35) and a significantly negative score in TR159 (−10.58) are obtained, which suggest potential antagonism. In contrast, when LB-100 is combined with DOX, there is a positive score in DT7 (1.62), indicating slight synergy; however, a negative score in TR159 (−5.12) points towards antagonistic interactions. The Bliss Independence model operates on a similar framework, asserting independent drug action. Here, the combination of LB-100 and adavosertib reveals negative scores in both DT7 (−0.79) and TR159 (−9.49), again highlighting a lack of synergy and the possibility of antagonism. Meanwhile, the LB-100 and DOX combination shows a positive score in DT7 (1.61), suggesting slight synergy, but the negative score in TR159 (−4.93) indicates a contrasting antagonistic effect. The Loewe Additivity model provides another perspective, which assumes an additive effect similar to that of a single agent. In this assessment, LB-100 combined with adavosertib results in a negative score in DT7 (−0.73) while yielding a positive score in TR159 (3.06), suggesting potential additivity or synergy in that context. For LB-100 and DOX, a strong positive score in DT7 (5.14) implies robust synergy or additivity, whereas the score in TR159 (0.03) remains very low, indicating weaker interaction. The Highest Single Agent (HSA) model becomes relevant when at least one of the drugs is ineffective, allowing for comparison between the combined effect and the most potent single agent. In the case of LB-100 with adavosertib, positive scores in both DT7 (3.67) and TR159 (5.92) reveal significant synergy. A similar favorable outcome is noted for the LB-100 and DOX combination, which shows positive scores in both DT7 (6.08) and TR159 (1.17), indicating notable synergistic potential.

### 3.4. Evaluating P-Glycoprotein Expression Modulation by LB-100, Adavosertib and Doxorubicin, and Their Combinations via Immunofluorescence Assay

**Figure 2:**
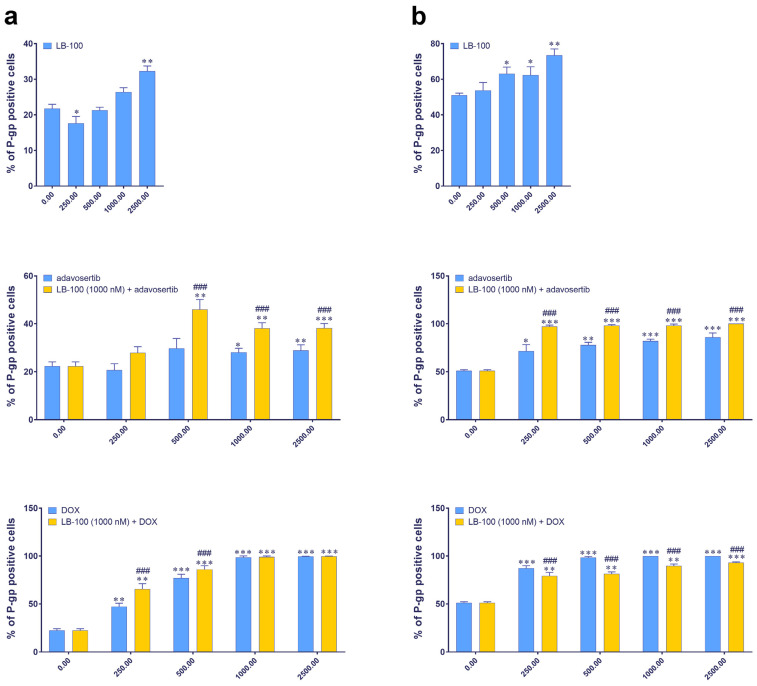
The expression of the P-gp in DT7 glioblastoma cells (**a**) and TR159 NSCLCs (**b**), evaluated following treatments with LB-100, adavosertib and doxorubicin (DOX), and a combination of 1 µM LB-100 with adavosertib and DOX. Assessments were performed using the ImageXpress PICO and corresponding software, CellReporterXpress version 2.8.2.669 (Molecular Devices). The graphs were generated in GraphPad Prism 8.0.2 and show the percentage of cells that tested positive for the P-gp antibody. Patient-derived cells were classified as inherently resistant if at least 20% of the cells were positive for the P-gp transporter. Data are presented as mean ± SEM with n = 4. Statistical significance compared to untreated control cells is as follows: * *p* < 0.05; ** *p* < 0.01; *** *p* < 0.001. The statistical significance of combined treatments compared to corresponding single treatments with adavosertib or DOX is indicated as ### *p* < 0.001.

We further examined the expression of P-gp, an MDR marker, in DT7 and TR159 cells after treatment with LB-100, adavosertib and DOX, and their combinations using the ImageXpress Pico Automated Cell Imaging System. Figure 2 shows that the baseline percentage of P-gp positive cells differs between the two cell lines: approximately 20% in DT7 cells and around 50% in TR159 cells (Figure 2). Both primary cell cultures were considered inherently resistant due to high basal expression of P-gp. All tested drugs significantly increased the percentage of P-gp positive cells in both DT7 and TR159 (Figure 2a,b).

When LB-100 was combined with adavosertib, it significantly increased the number of P-gp positive cells in both DT7 (Figure 2a) and TR159 (Figure 2b). A similar effect was observed when LB-100 was combined with DOX in DT7 cells (Figure 2a). However, in the case of TR159, combining LB-100 with DOX significantly reduced the percentage of P-gp positive cells compared to DOX alone (Figure 2b).

### 3.5. LB-100 Decreases P-Glycoprotein Expression Multidrug-Resistant Cancer Cells

**Figure 3:**
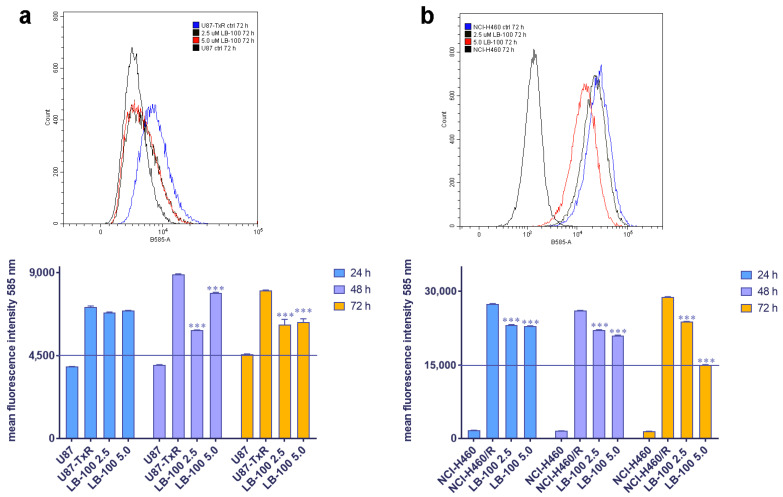
The time- and dose-dependent effects of LB-100 on P-gp expression in multidrug-resistant U87-TxR glioblastoma cells (**a**) and NCI-H460/R NSCLCs (**b**). The corresponding sensitive cells, U87 and NCI-H460, were used as reference controls for P-gp expression in the multidrug-resistant cells. Flow cytometry profiles representing results obtained after 72 h were generated using the CytoFLEX flow cytometer and the CytExpert software (Beckman Coulter, Indianapolis, IN, USA). The graphs were created in GraphPad Prism 8.0.2 software. Each sample included at least 10,000 events. The data are presented as mean ± SEM, with statistical significance indicated compared to the untreated control U87-TxR and NCI-H460/R cells: *** *p* < 0.001.

We then assessed the impact of LB-100 on P-gp expression in MDR cancer cell lines of glioblastoma and NSCLC origin, U87-TxR and NCI-H460/R, respectively. These cell lines were treated with 2.5 µM and 5.0 µM LB-100 for 24 h, 48 h and 72 h. Flow cytometry results, depicted in Figure 3, revealed that LB-100 significantly reduced P-gp expression in U87-TxR after 48 h and 72 h (Figure 3a). Interestingly, this effect was not dose-dependent, as the 2.5 µM concentration demonstrated a more substantial effect than 5 µM (Figure 3a). In contrast, for NCI-H460/R, LB-100 led to a significant decrease in P-gp expression across all time points (Figure 3b). This effect was both time- and dose-dependent, with the most pronounced reduction in P-gp expression observed with 5.0 µM LB-100 at 72 h (Figure 3b).

### 3.6. LB-100 Affects the Expression of Genes That Contribute to Drug Resistance in Cancer Cells

**Figure 4:**
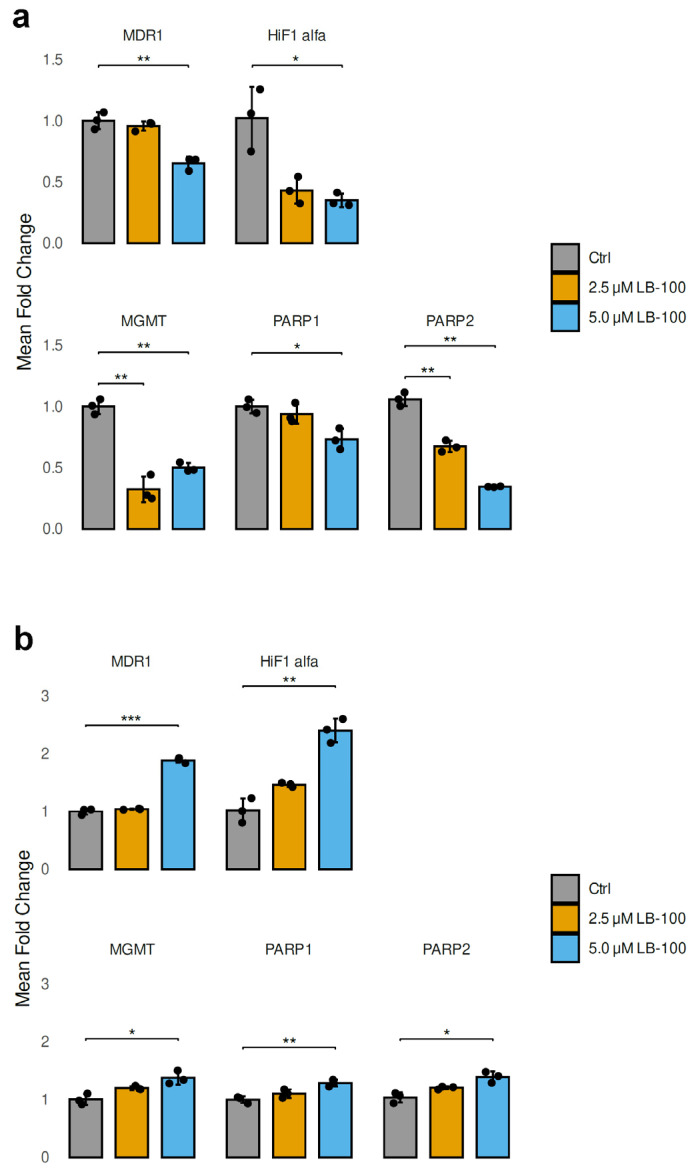
Relative mRNA expression levels of drug resistance-related genes (*MDR1*, *HIF-1α*, *MGMT*, *PARP1* and *PARP2*) analyzed following a 24 h treatment with LB-100 in U87-TxR (**a**) and NCI-H460/R (**b**) cells. ACTB was used for normalization (n = 3). The graphs and figures were generated in R—statistical software with the ggplot2 package (Version 3.5.1). The data are presented as mean ± SEM with n = 3. Statistical significance compared to untreated control cells is as follows: * *p* < 0.05; ** *p* < 0.01; *** *p* < 0.001.

In our study, we analyzed the mRNA expression levels of genes contributing to drug resistance in cancer cells after treatment with 2.5 µM and 5.0 µM LB-100 for 24 h in U87-TxR and NCI-H460/R cell lines (Figure 4). In addition to *MDR1* (*ABCB1*), which encodes P-gp, we also examined *HIF-1α*, a transcription factor that regulates genes associated with energy metabolism and angiogenesis. Furthermore, we investigated *MGMT* (O6-methylguanine-DNA methyltransferase), *PARP1* and *PARP2* (poly [ADP-ribose] polymerase), all of which are crucial for DNA repair.

The treatment with LB-100 resulted in a significant decrease in the mRNA levels of *MDR1*, *HIF-1α*, *MGMT*, *PARP1* and *PARP2* in U87-TxR cells, as illustrated in Figure 4a. This reduction was particularly pronounced at a concentration of 5.0 µM, with the exception of MGMT, which did not follow the same trend (Figure 4a). In contrast, the mRNA levels of *MDR1*, *HIF-1α*, *MGMT*, *PARP1* and *PARP2* exhibited a marked increase in NCI-H460/R cells when treated with 5.0 µM LB-100. The treatment with 2.5 µM LB-100 did not produce any significant changes when compared to the untreated cells (Figure 4b).

### 3.7. LB-100 Is Not a P-Glycoprotein Substrate

The SwissADME analysis, shown in Figure S6 and illustrated as a “boiled egg”, indicated that LB-100 can be absorbed by the human intestine. However, LB-100 does not cross the blood–brain barrier and is not a substrate for P-gp. Furthermore, LB-100 does not inhibit CYP proteins, a superfamily of enzymes involved in drug metabolism. The data in Figure S7 show that LB-100 has a minimal cytotoxic effect on U87-TxR and NCI-H460/R cells.

To further confirm that LB-100 is not a substrate for P-gp, a Rho123 accumulation assay was conducted. U87-TxR and NCI-H460/R cells expressing P-gp were treated with LB-100 at concentrations of 2.5, 5 and 10 µM for 30 min to assess any direct interaction with P-gp (Figures S8 and S9). The results showed that LB-100 did not alter the accumulation of Rho123 in either U87-TxR or NCI-H460/R cells.

### 3.8. LB-100 Sensitizes Multidrug-Resistant Cancer Cells to Doxorubicin

**Figure 5:**
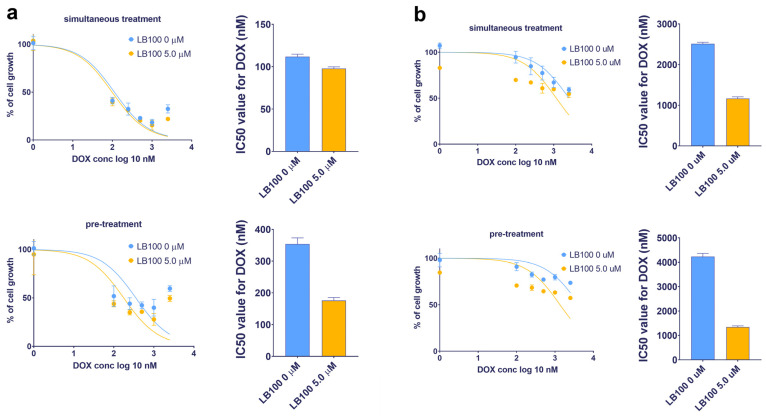
Reversal of doxorubicin (DOX) resistance by LB-100 in simultaneous and subsequent treatment schedules in U87-TxR glioblastoma (**a**) and NCI-H460/R NSCLC (**b**) cells. The cell growth was compared between DOX administered alone and in combination with LB-100. Both treatment schedules lasted for 72 h. In the subsequent treatment schedule, DOX was applied for 48 h, following the administration of LB-100 24 h earlier. The results were assessed by MTT, while the graphs were created in GraphPad Prism 8.0.2.

Considering the potential of LB-100 to suppress P-gp expression, its ability to reverse DOX resistance—which occurs because DOX is a substrate of P-gp—was examined in U87-TxR and NCI-H460/R cell lines (Table 3 and Figure 5). The effects of LB-100 in combination with DOX were assessed using both simultaneous and subsequent treatment schedules after 72 h, as determined by the MTT assay. The results indicated that LB-100 enhanced the efficacy of DOX in U87-TxR cells, with a more pronounced effect observed in the subsequent treatment schedule. Specifically, pre-treatment with 5.0 µM LB-100 resulted in a 2-fold decrease in the IC50 value for DOX (Figure 5a and Table 3). In contrast, simultaneous administration of LB-100 and DOX produced a similar effect on cell growth as DOX treatment alone (Figure 5a and Table 3). In the NCI-H460/R cell line, a simultaneous treatment with 5.0 µM LB-100 also led to a 2-fold decrease in the IC50 value for DOX; however, a 3-fold decrease was observed with the subsequent treatment schedule (Figure 5b and Table 3). A similar trend was noted with a lower concentration of 2.5 µM LB-100 in both MDR cancer cell lines (Table 3).

### 3.9. LB-100 Increases Doxorubicin Accumulation in Multidrug-Resistant Cancer Cells

**Figure 6:**
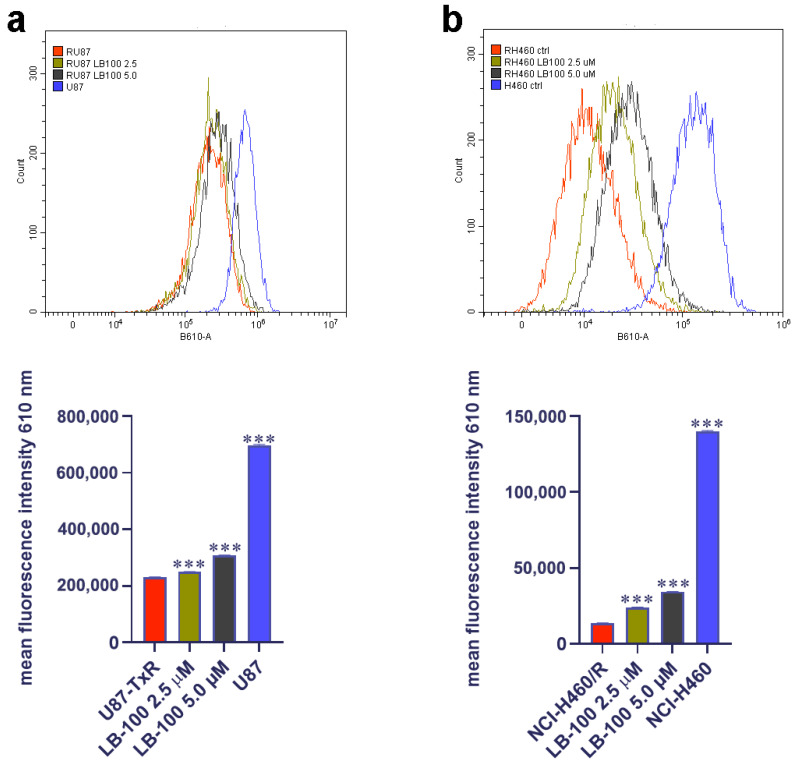
Doxorubicin (DOX) accumulation study after LB-100 treatment in U87-TxR glioblastoma (**a**) and NCI-H460/R NSCLCs (**b**). Flow cytometry profiles were generated using the CytoFLEX flow cytometer and the CytExpert software (Beckman Coulter, Indianapolis, IN, USA). The graphs were created in GraphPad Prism 8.0.2 software. Each sample included at least 10,000 events. The data are presented as mean ± SEM, with statistical significance indicated compared to untreated control U87-TxR and NCI-H460/R cells: *** *p* < 0.001.

After exploring the impact of LB-100 on P-gp expression and its potential to reverse DOX resistance in MDR cancer cells, we assessed its capacity to enhance DOX accumulation in these cells. To achieve this, we treated the cells with 2.5 µM and 5.0 µM of LB-100 for 72 h and then assessed the accumulation of 10 µM DOX (Figure 6). Our findings revealed that both concentrations of LB-100 significantly increased DOX accumulation in U87-TxR cells (Figure 6a), with an even more pronounced effect observed in NCI-H460/R cells (Figure 6b).

### 3.10. The Impact of LB-100 and Doxorubicin on the Cell Cycle in Multidrug-Resistant Cancer Cells

Next, we examined the effects of the combination of LB-100 and DOX on the cell cycle kinetics in U87-TxR and NCI-H460/R cells. Figure S10 illustrates the impact of 2.5 µM LB-100 on U87-TxR cells, which increased the percentage of cells in the G2/M phase after 72 h. Treatment with 250 nM DOX for 48 h led to a significant decrease in the G1 phase, followed by an arrest in the G2/M phase, without an increase in the portion of dead cells in the subG0 phase (Figure S10). Pre-treatment with LB-100 increased the percentage of U87-TxR cells in the subG0 phase and decreased the portion of cells in the G2/M phase compared to DOX treatment alone (Figure S10). Our findings indicate that 5.0 µM LB-100 increased the number of dead cells in the subG0 phase in NCI-H460/R cells (Figure S11). Treatment with 500 nM DOX alone caused a significant shift of NCI-H460/R cells into the G2/M phase, followed by an increase in the subG0 phase (Figure S11). In the subsequent treatment schedule, LB-100 further increased the percentage of NCI-H460/R cells in the G2/M phase compared to treatment with DOX alone (Figure S11).

### 3.11. The Impact of LB-100 and Doxorubicin on the Induction of DNA Damage

We compared the impact of LB-100, DOX and their combination on DNA damage induction (Figure S12). To assess DNA damage in U87-TxR and NCI-H460/R cells, we measured the expression levels of pH2A.X histone, a DNA double-stranded breaks marker, after the subsequent treatment schedule described earlier. Our results showed that DOX at 250 nM and 500 nM caused significant DNA damage in both U87-TxR and NCI-H460/R cells, respectively (Figure S12). On the other hand, LB-100 slightly decreased pH2A.X expression at 2.5 µM and 5.0 µM in U87-TxR and NCI-H460/R cells, respectively (Figure S12). LB-100 in both MDR cancer cell lines slightly suppressed the effect of DOX (Figure S12).

## 4. Discussion

LB-100, a PP2A inhibitor, can counteract cancer cell senescence, promote the differentiation of progenitor cells, enhance drug penetration and induce mitotic catastrophe and cell death [12]. In this study, we explored the effects of LB-100 in combination with adavosertib and doxorubicin (DOX) in patient-derived cell cultures of glioblastoma and NSCLCs. Additionally, we conducted studies using MDR cellular models of glioblastoma and NSCLCs to investigate the potential mechanisms behind the effects of LB-100 in combination with other drugs, using DOX as a reference for this study.

Our results from multiple applications of LB-100 in combination with adavosertib and DOX in patient-derived glioblastoma and NSCLCs align with findings from previous research that demonstrated synergy between adavosertib and LB-100 in various models of colon cancer, pancreatic cancer and cholangiocarcinoma [16]. However, single applications of this combined treatment, evaluated through an ex vivo immunofluorescence assay and the SynergyFinder+ online tool, indicated that LB-100 enhances the effects of adavosertib and DOX but without synergistic interaction.

The combination of LB-100 and adavosertib induces replication stress, which leads to premature mitosis and cell death. Prior studies have shown that LB-100 downregulates DNA repair signaling and deregulates several mitotic proteins, resulting in replication stress and mitotic catastrophe [19,41]. Additionally, adavosertib drives cells with unrepaired DNA damage into mitosis by inhibiting WEE1, leading to mitotic catastrophe [42]. Thus, the synergistic effect of LB-100 and adavosertib stems from their complementary impacts on cell cycle regulation, DNA damage repair and apoptosis. However, our experimental setting indicates that the combination must be applied multiple times to achieve synergistic interaction.

DOX intercalates into DNA, disrupts DNA replication and transcription and inhibits topoisomerase II, inducing DNA double-strand breaks [43]. Since LB-100 downregulates DNA repair signaling, it may inhibit the repair of DOX-induced DNA damage, forcing cells with damaged DNA into mitosis and ultimately causing cell death. Similar to the LB-100 and adavosertib combination, our results indicate that achieving synergistic interaction between LB-100 and DOX also requires multiple applications.

We analyzed the expression of the MDR marker P-gp in patient-derived cells of glioblastoma and NSCLCs after combined treatment with LB-100 and adavosertib/DOX, using a modified immunoassay developed in our laboratory [33]. LB-100 alone tended to increase P-gp expression, particularly at higher concentrations. The PP2A modulates P-gp expression by influencing signaling pathways, transcription factors and posttranslational modifications associated with P-gp expression and activity [44]. As a result, dysregulation of PP2A activity may lead to elevated P-gp expression through various mechanisms. When LB-100 was added to adavosertib, there was a further increase in P-gp expression compared to adavosertib alone in both primary cell cultures. However, the combined treatment of LB-100 and DOX presented different outcomes. In glioblastoma primary cells, LB-100 further increased P-gp expression at lower concentrations of DOX. In contrast, in NSCLC primary cells, LB-100 resulted in a significant decrease in P-gp expression compared to DOX alone.

The ability of LB-100 to modify P-gp levels was examined at the transcriptional and protein levels in glioblastoma and NSCLC MDR models, which exhibit overexpression of P-gp, compared to their sensitive counterpart cells. Treatment with LB-100 led to a time-dependent reduction in P-gp expression in both MDR models. Notably, LB-100 induced a cell type-dependent effect on *ABCB1* gene expression, which encodes P-gp. Specifically, LB-100 decreased *HIF1α* mRNA expression in MDR glioblastoma U87-TxR cells. The inhibition of PP2A by LB-100 may increase the phosphorylation of proteins involved in regulating HIF-1α degradation [45], leading to a reduction in HIF-1α levels, subsequently suppressing its transcriptional activation of target genes, including *ABCB1*. The observed decrease in *ABCB1* mRNA expression after 24 h of treatment with LB-100 is followed by a decline in P-gp levels after 48 h and 72 h.

Our findings revealed increased *ABCB1* and *HIF1α* mRNA expression following LB-100 treatment in MDR NSCLC NCI-H460/R cells. HIF-1α is a transcription factor tightly regulated at the transcriptional level by multiple signaling pathways. The PI3K-Akt-mTOR and ERK signaling pathways promote *HIF-1α* mRNA expression [46,47]. PP2A dephosphorylates and inactivates Akt, mTOR and the ERK signaling pathway [48]. Thus, the inhibition of PP2A hyperactivates mTOR and maintains ERK activation, leading to enhanced *HIF-1α* mRNA expression. HIF-1α binds directly to hypoxia response elements (HREs) in the promoter of the *ABCB1* gene, driving its transcription [49]. Despite the increase in *ABCB1* gene transcription after LB-100 treatment, P-gp expression shows a concentration-dependent decrease maintained over time, indicating that the post-translational regulation of P-gp is also affected. It is known that the activity and stability of P-gp are influenced by its phosphorylation status. The reduction in P-gp protein levels by LB-100 may result from altered protein stability, likely due to PP2A inhibition disrupting the phosphorylation states involved in the turnover or degradation of P-gp [44,50] suggesting that while *ABCB1* transcription is upregulated, along with its transcriptional regulator *HIF-1α*, P-gp becomes less stable or is degraded more rapidly, possibly due to changes in cellular stress or protein quality control mechanisms activated by PP2A inhibition.

HIF-1α also plays a crucial role in regulating the transcription of the *MGMT* gene, which encodes the DNA repair protein O6-methylguanine-DNA-methyl-transferase [51]. Consequently, the LB-100-induced decrease in *HIF-1α* mRNA expression was followed by reduced *MGMT* gene transcription in MDR glioblastoma cells. This reduction in *MGMT* transcription levels, prompted by LB-100, could be advantageous for glioblastoma patients, as the absence of *MGMT* expression is regarded as a favorable prognostic indicator for temozolomide-treated glioblastoma patients [52].

LB-100 can influence DNA repair processes and disrupt several proteins that are involved in cell-cycle checkpoints [19,41]. This effect may increase the dependence on DNA damage repair proteins such as poly (ADP-ribose) polymerases (PARPs), potentially altering their expression levels. Our results showed decreased gene expression of *PARP1* and *PARP2* in MDR glioblastoma cells, while MDR NSCLCs exhibited increased mRNA expression of these genes.

We also conducted a cell cycle analysis and assessed DNA damage by detecting double-strand breaks to explore whether changes in the cell cycle or the induction of DNA damage contribute to the increased efficacy of DOX when combined with LB-100. Our findings indicated that adding LB-100 does not change the cell cycle dynamics or induce DNA damage compared to DOX alone.

P-gp plays a critical role in eliminating various anticancer drugs, including DOX [53]. Since LB-100 has shown potential in suppressing P-gp expression, we hypothesized that combining LB-100 with DOX could be beneficial in sensitizing MDR cancer cells. Therefore, we assessed the cytotoxicity of simultaneous and subsequent combined treatments. In U87-TxR cells, only the subsequent administration of LB-100 enhanced the cytotoxicity of DOX. In NCI-H460/R cells, LB-100 improved the efficacy of DOX with both treatment schedules, although the subsequent treatment demonstrated a more pronounced effect. Our findings highlight the importance of the sequence, in which LB-100 is administered alongside other drugs. Additionally, a DOX accumulation assay conducted after 72 h with LB-100 revealed a concentration-dependent increase in DOX accumulation in both MDR cancer cell models. This result aligns with the observed impact of LB-100 on DOX efficacy in simultaneous and subsequent treatments and could also be due to LB-100 ability to enhance drug penetration [12]. The Rho123 accumulation assay demonstrated that LB-100 does not affect P-gp activity, while the SwissADME analysis indicated that LB-100 is not a P-gp substrate. Therefore, our findings suggest that LB-100 sensitizes MDR cancer cells by altering P-gp expression.

The impact of LB-100 on P-gp expression and activity has not been studied previously. According to the SwissADME tool, LB-100 cannot penetrate the BBB, which includes P-gp as a key component. While the SwissADME tool offers predictive insights, validating these findings through experimental studies is essential to determine whether LB-100 can indeed cross the BBB. Additionally, this tool suggests that LB-100 is not a substrate for P-gp. To confirm this, we used the Rho123 accumulation assay in MDR cancer cells expressing P-gp, which supported the prediction.

Mechanistically, LB-100 reduces P-gp expression in MDR cancer cells. However, the transcriptional pattern of the *ABCB1* gene varies between MDR glioblastoma and NSCLCs, suggesting that regulation may occur at the post-transcriptional level. *HIF-1α*, a transcriptional regulator of *ABCB1*, also exhibited a different pattern in MDR glioblastoma and NSCLCs that mirrors *ABCB1* expression.

Furthermore, SwissADME shows that LB-100 does not interact with enzymes involved in drug metabolism, making it a promising candidate for combination therapy. The impact of LB-100 on P-gp expression is most significant after 72 h, which correlates with increased DOX accumulation and enhanced sensitivity of MDR cancer cells to DOX treatment. Notably, LB-100 does not appear to affect DNA damage or disrupt the cell cycle, indicating that MDR cancer cells possess robust DNA repair mechanisms that may counteract the typical effects of LB-100 on DNA damage induction and alterations in cell cycle dynamics.

## 5. Conclusions

In summary, combining LB-100 with adavosertib and DOX is effective for glioblastoma and NSCLCs, but multiple applications are required to achieve synergistic effects. LB-100 demonstrates potential in addressing drug resistance in MDR cancers by reducing P-gp levels while affecting key regulatory pathways and altering transcriptional dynamics of genes associated with treatment response. The observed effects on *HIF-1α* and *MGMT* gene expression suggest that LB-100 may enhance the efficacy of approved therapies like temozolomide, commonly used for glioblastoma therapy. LB-100 effectively enhances the accumulation of DOX and its cytotoxicity in MDR cancer cells, particularly when administered in a subsequent treatment. Our findings indicate that LB-100 alters P-gp expression, thereby improving the efficacy of DOX. Combining LB-100 with anticancer drugs is a promising strategy for overcoming drug resistance in cancer therapy.

## Figures and Tables

**Table 1:** The effects of LB-100 and adavosertib/doxorubicin combinations on glioblastoma (DT7) and NSCLC (TR159) primary cell cultures expressed with IC50 values (nM) determined using non-linear regression.

	DT7	TR159
LB-100	10,808 ± 365	931 ± 17
Adavosertib	4930 ± 103	374 ± 7
Adavosertib + LB-100 ^1^	4467 ± 111 ^ns^	248 ± 6 *
Adavosertib + LB-100 ^2^	3505 ± 81 *	240 ± 8 *
Adavosertib + LB-100 ^3^	2417 ± 47 **	238 ± 7 *
Doxorubicin	582 ± 13	91 ± 2
Doxorubicin + LB-100 ^1^	469 ± 8 *	86 ± 1 ^ns^
Doxorubicin + LB-100 ^2^	443 ± 10 *	89 ± 1 ^ns^
Doxorubicin + LB-100 ^3^	394 ± 7 **	88 ± 1 ^ns^

^1^ DT7 cells were treated with 500 nM of LB-100, while TR159 cells were treated with 250 nM of LB-100. ^2^ DT7 cells were treated with 1000 nM of LB-100, while TR159 cells were treated with 500 nM of LB-100. ^3^ DT7 cells were treated with 2500 nM of LB-100, while TR159 cells were treated with 1000 nM of LB-100. Statistically significant effect of combinations in comparison with adavosertib/doxorubicin alone: * *p* < 0.05, ** *p* < 0.01, ns = non-significant.

**Table 2:** The nature of LB-100 interaction with adavosertib/doxorubicin in glioblastoma (DT7) and NSCLC (TR159) primary cell cultures evaluated by the SynergyFinder+ online tool.

	DT7				TR159			
Synergy score in different reference models	**ZIP ^1^**	**Bliss ^2^**	**Loewe ^3^**	**HSA ^4^**	**ZIP ^1^**	**Bliss ^2^**	**Loewe ^3^**	**HSA ^4^**
LB-100 + adavosertib	−0.35	−0.79	−0.73	3.67 ^SYN^	−10.58	−9.49	3.06 ^SYN^	5.92 ^SYN^
LB-100 + doxorubicin	1.62	1.61	5.14 ^SYN^	6.08 ^SYN^	−5.12	−4.93	0.03	1.17 ^SYN^

^1^ ZIP (Zero Interaction Potency)—this model assumes that drugs work independently and do not interact with each other when combined. However, this assumption does not hold for the combinations we tested. ^2^ Bliss Independence—this model also assumes that both drugs act independently and do not interfere with each other. Again, this assumption does not apply to the combinations we tested. ^3^ Loewe Additivity—this model is only applicable to drugs that maintain a constant potency ratio, which is not the case with the drugs we investigated. ^4^ HSA (Highest Single Agent)—this model is suitable for drug combinations in which at least one of the drugs is inactive across all tested concentrations. In our study, LB-100 can be considered inactive regarding cytotoxicity. ^SYN^ indicates synergistic interaction in specific reference models.

**Table 3:** The impact of LB-100 on doxorubicin efficacy in combined simultaneous and subsequent treatments expressed as IC50 values of doxorubicin (nM).

U87-TxR	**LB-100 0 µM**	**LB-100 2.5 µM**	**LB-100 5.0 µM**
Simultaneous treatment	113 ± 3	96 ± 2	95 ± 3
Subsequent treatment	354 ± 19	197 ± 18	171 ± 11
NCI-H460/R	**LB-100 0 µM**	**LB-100 2.5 µM**	**LB-100 5.0 µM**
Simultaneous treatment	2592 ± 63	1436 ± 29	1168 ± 40
Subsequent treatment	4169 ± 147	1896 ± 76	1347 ± 44

## Data Availability

The raw data supporting the conclusions of this article will be made available by the authors on request.

## Supplementary Materials

The following supporting information can be downloaded at: https://www.mdpi.com/article/10.3390/pharmaceutics17020189/s1, Figure S1: Image analysis of ABCB1-overexpressing cells in DT7 glioblastoma and TR159 NSCLC cultures; Figure S2: Multiple reference models (ZIP, Bliss, Loewe, and HAS) were generated using SynergyFinder+ based on the Dose Response Matrix derived from the combination treatment of LB-100 and adavosertib (ADAVO) in glioblastoma patient-derived cells (DT7); Figure S3: Multiple reference models (ZIP, Bliss, Loewe, and HAS) were generated using SynergyFinder+ based on the Dose Response Matrix derived from the combination treatment of LB-100 and doxorubicin (DOX) in glioblastoma patient-derived cells (DT7); Figure S4: Multiple reference models (ZIP, Bliss, Loewe, and HAS) were generated using SynergyFinder+ based on the Dose Response Matrix derived from the combination treatment of LB-100 and adavosertib (ADAVO) in NSCLC patient-derived cells (TR159); Figure S5: Multiple reference models (ZIP, Bliss, Loewe, and HAS) were generated using SynergyFinder+ based on the Dose Response Matrix derived from the combination treatment of LB-100 and doxorubicin (DOX) in NSCLC patient-derived cells (TR159); Figure S6: The "boiled-egg" plot — representing the Brain or Intestinal Estimated Permeation Predictive Model — was constructed for LB-100 utilizing the SwissADME online tool; Figure S7: The impact of LB-100 on multidrug-resistant (MDR) cancer cell lines, glioblastoma U87-TxR and non-small cell lung carcinoma NCI-H460/R; Figure S8: Rhodamine 123 accumulation after LB-100 treatment was evaluated in multidrug-resistant U87-TxR (RU87) glioblastoma cells, which overexpress P-glycoprotein (P-gp); Figure S9: Rhodamine 123 accumulation after LB-100 treatment was evaluated in multidrug-resistant NCI-H460/R (RH460) non-small cell lung carcinoma cells, which overexpress P-glycoprotein (P-gp); Figure S10: The cell cycle distribution of multidrug-resistant U87-TxR glioblastoma cells was evaluated via propidium iodide (PI) staining following combined treatment with LB-100 and doxorubicin (DOX); Figure S11: The cell cycle distribution of multidrug-resistant NCI-H460/R non-small cell lung carcinoma cells was evaluated via propidium iodide (PI) staining following combined treatment with LB-100 and doxorubicin (DOX); Figure S12: DNA damage was assessed by quantifying the expression levels of phospho-histone 2A.X (pH2A.X) in multidrug-resistant U87-TxR (RU87) glioblastoma and NCI-H460/R non-small cell lung carcinoma cells.
