# Supplementary material for: LB-100 Enhances Drugs Efficacy Through Inhibition of P-Glycoprotein Expression in Multidrug-Resistant Glioblastoma and Non-Small Cell Lung Carcinoma Cellular Models
Source: Pharmaceutics. 2025 Feb 4;17(2):189. doi: 10.3390/pharmaceutics17020189 (PMC11859366; doi:10.3390/pharmaceutics17020189)
Supplement: Supplementary file 1 [file pharmaceutics-17-00189-s001.zip › pharmaceutics-3396719-supplementary.pdf]

Supplementary Material

LB-100 Enhances Drugs Efficacy through Inhibition of P-glycoprotien Expression in Multidrug-Resistant Glioblastoma and Non-Small Cell Lung Carcinoma Cellular Models

Ana Podolski-Renić [1,†], Margarita Chigriai [2,†], Sofija Jovanović Stojanov [1], Marija Grozdanić [1], Ema Lupšić [1], Igor Nikolić [3, 4], Miodrag Dragoj [1], Jelena Dinić [1], and Milica Pešić [1,*]

[1] Institute for Biological Research "Siniša Stanković" - National Institute of the Republic of Serbia, University of Belgrade, Despota Stefana 142, 11108 Belgrade, Serbia; ana.podolski@ibiss.bg.ac.rs (A.P-R.); sofija.jovanovic@ibiss.bg.ac.rs (S.J.S.); marija.grozdanic@ibiss.bg.ac.rs (M. G.); ema.lupsic@ibiss.bg.ac.rs (E.L.) miodrag.dragoj@ibiss.bg.ac.rs (M.D.); jelena.dinic@ibiss.bg.ac.rs (J.D.); camala@ibiss.bg.ac.rs (M. P.)

[2] Advitam Laboratory, Mihaila Suskalovica 13, 11030, Belgrade, Serbia; chigriai@advitamlab.com (M.C.)

[3] Clinic for Neurosurgery, Clinical Center of Serbia, Pasterova 2, 11000 Belgrade, Serbia; igor.nikolic@med.bg.ac.rs (I.N.)

[4] School of Medicine, University of Belgrade, Doktora Subotića 8, 11000 Belgrade, Serbia; igor.nikolic@med.bg.ac.rs (I.N.)

† These authors contributed equally to this work.

* Correspondence: camala@ibiss.bg.ac.rs (M. P.)

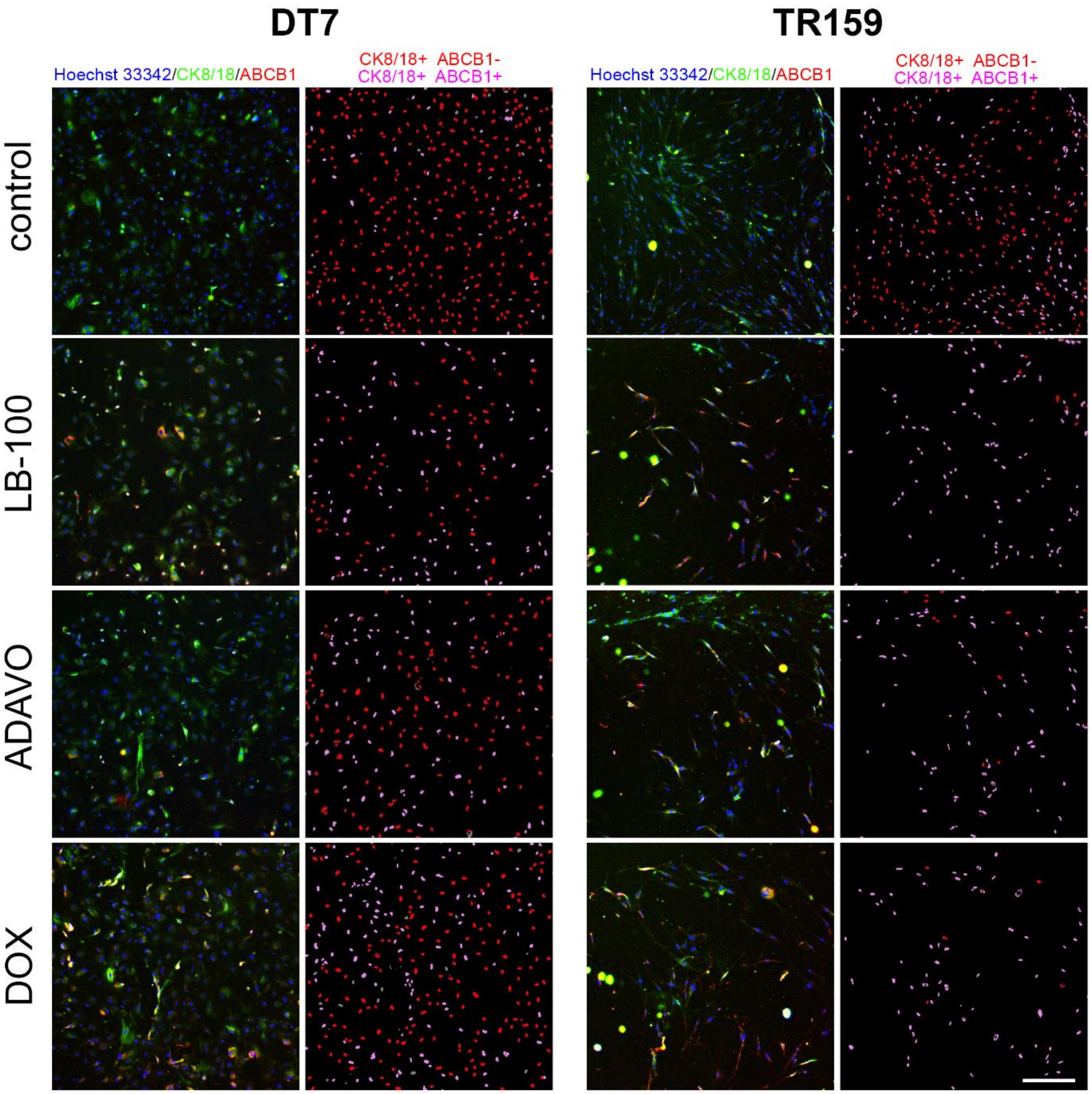

Figure S1. Image analysis of ABCB1-overexpressing cells in DT7 glioblastoma and TR159 NSCLC cultures. Fluorescence micrographs of DT7 and TR159 cultures, grown in 384-well plates, fixed, and stained with Hoechst 33342 (blue), anti-CK8/18-Alexa Fluor 488 (green), and anti-ABCB1-Alexa Fluor 555 (red), are shown in the left panels. Images were captured using the ImageXpress® Pico (Molecular Devices®). The right panels display cell scoring analysis, which categorizes DT7 and TR159 populations based on ABCB1 expression: low expression (CK8/18+/ABCB1-; red) and high expression (CK8/18+/ABCB1+; pink). Image analysis was performed using the Multi-Wavelength Cell Scoring Analysis Protocol in CellReporterXpress® software. Scale bar = 500 μm.

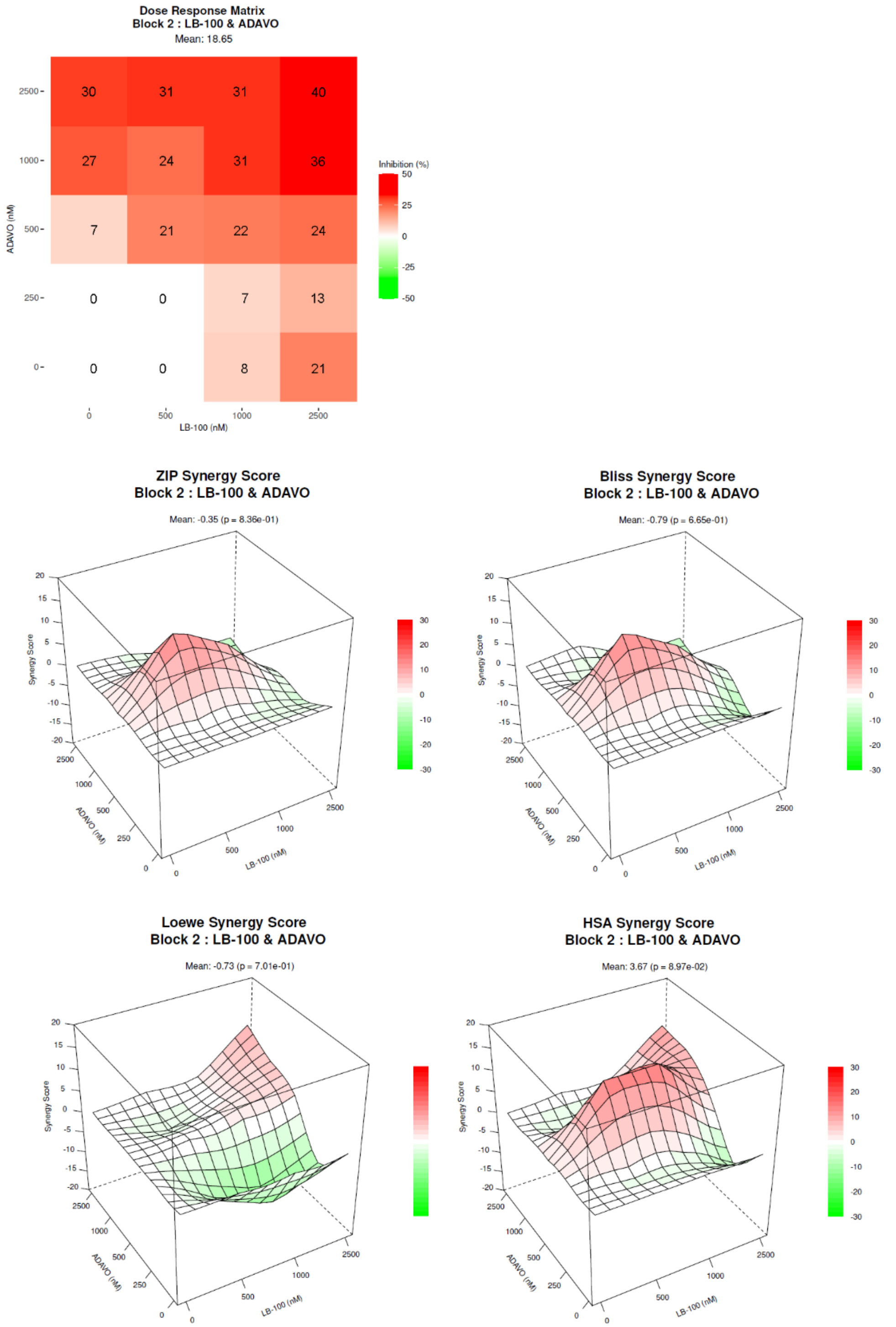

Figure S2. Multiple reference models (ZIP, Bliss, Loewe, and HAS) were generated using SynergyFinder+ based on the Dose Response Matrix derived from the combination treatment of LB-100 and adavosertib (ADAVO) in glioblastoma patient-derived cells (DT7). Cytotoxic effects were evaluated through an immunofluorescence assay, as detailed in the Materials and Methods section. Positive Synergy Scores, indicated in red, reflect significant synergistic interactions, whereas negative Synergy Scores, shown in green, denote antagonistic effects.

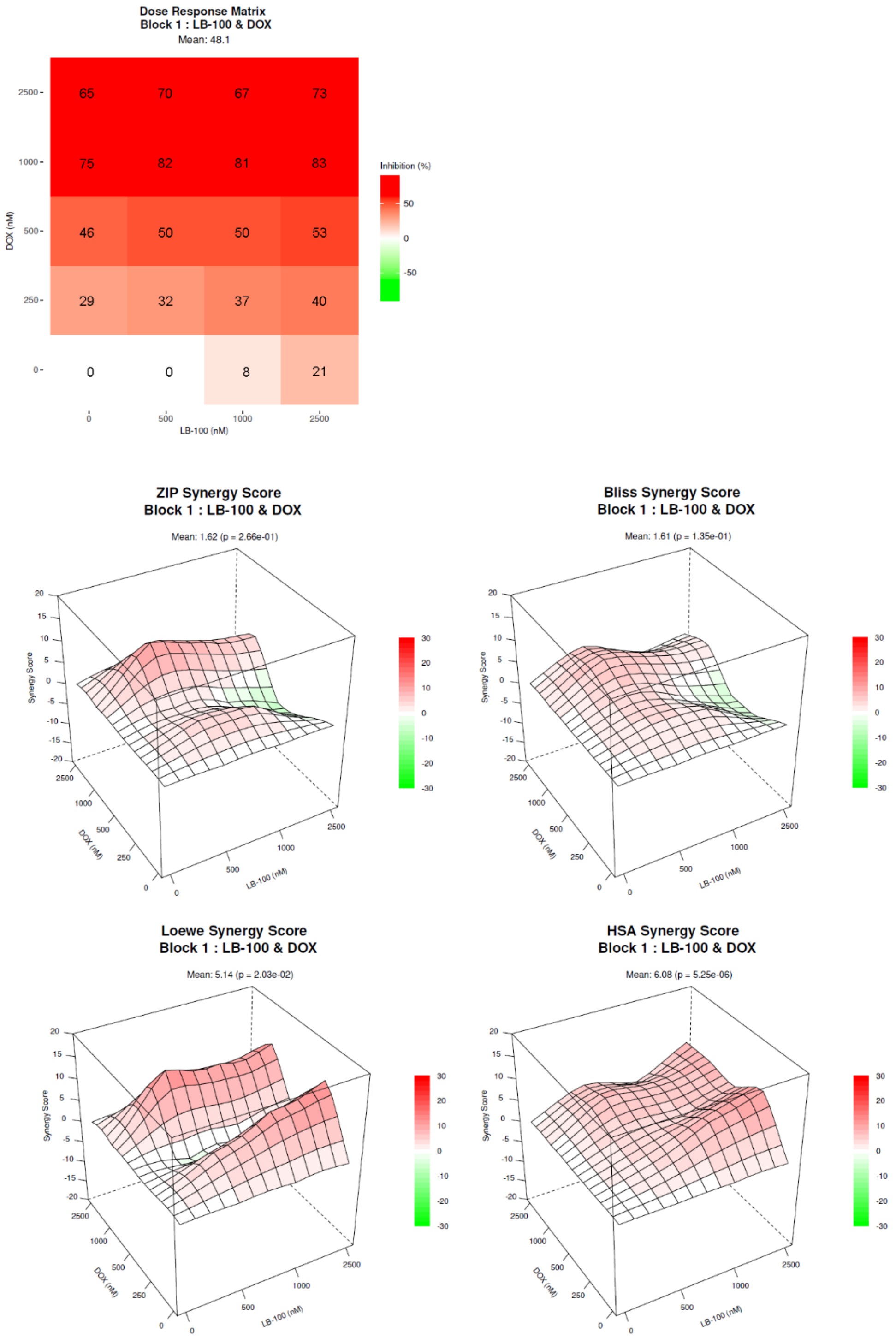

Figure S3. Multiple reference models (ZIP, Bliss, Loewe, and HAS) were generated using SynergyFinder+ based on the Dose Response Matrix derived from the combination treatment of LB-100 and doxorubicin (DOX) in glioblastoma patient-derived cells (DT7). Cytotoxic effects were evaluated through an immunofluorescence assay, as detailed in the Materials and Methods section. Positive Synergy Scores, indicated in red, reflect significant synergistic interactions, whereas negative Synergy Scores, shown in green, denote antagonistic effects.

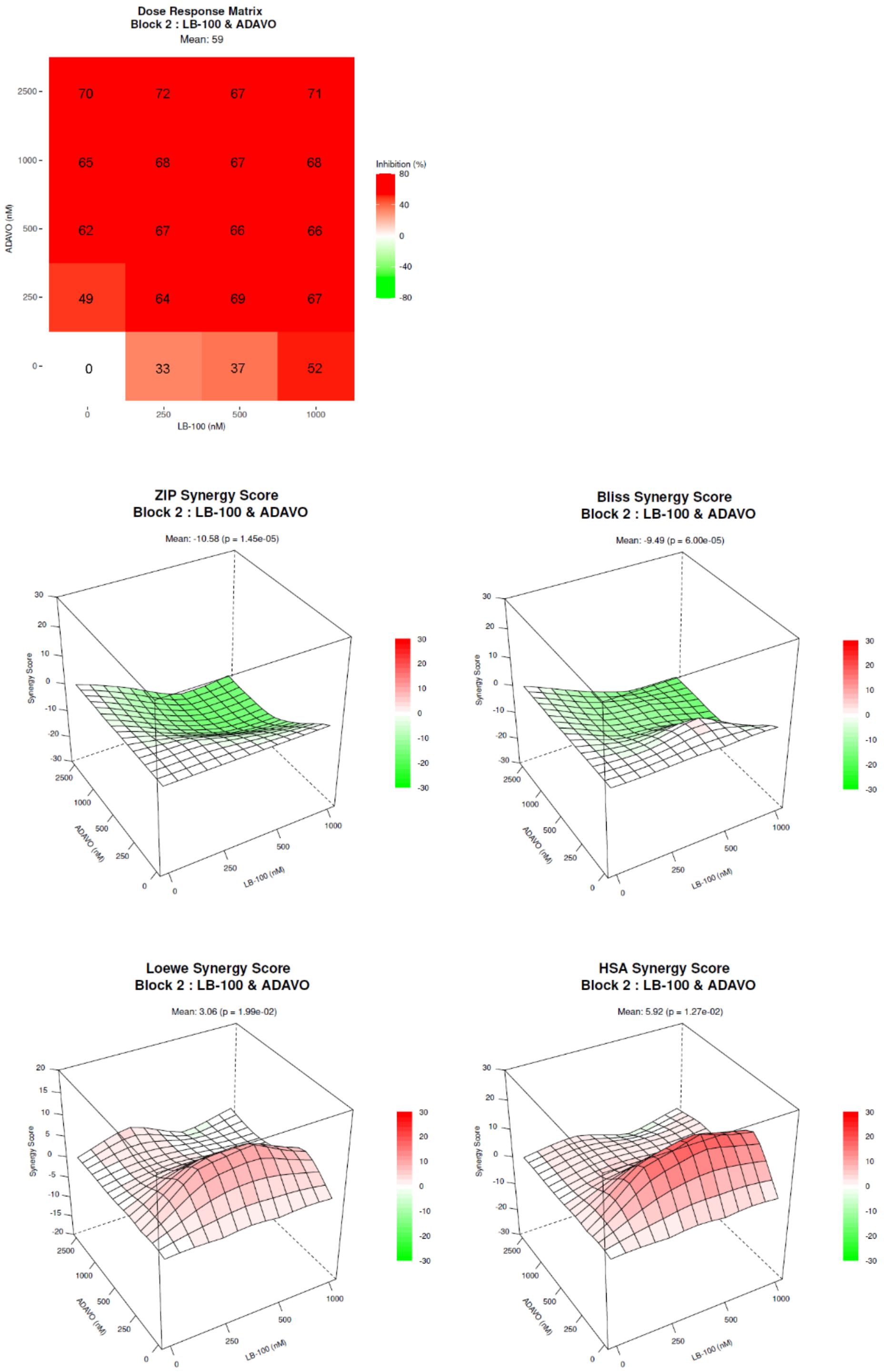

Figure S4. Multiple reference models (ZIP, Bliss, Loewe, and HAS) were generated using SynergyFinder+ based on the Dose Response Matrix derived from the combination treatment of LB-100 and adavosertib (ADAVO) in NSCLC patient-derived cells (TR159). Cytotoxic effects were evaluated through an immunofluorescence assay, as detailed in the Materials and Methods section. Positive Synergy Scores, indicated in red, reflect significant synergistic interactions, whereas negative Synergy Scores, shown in green, denote antagonistic effects.

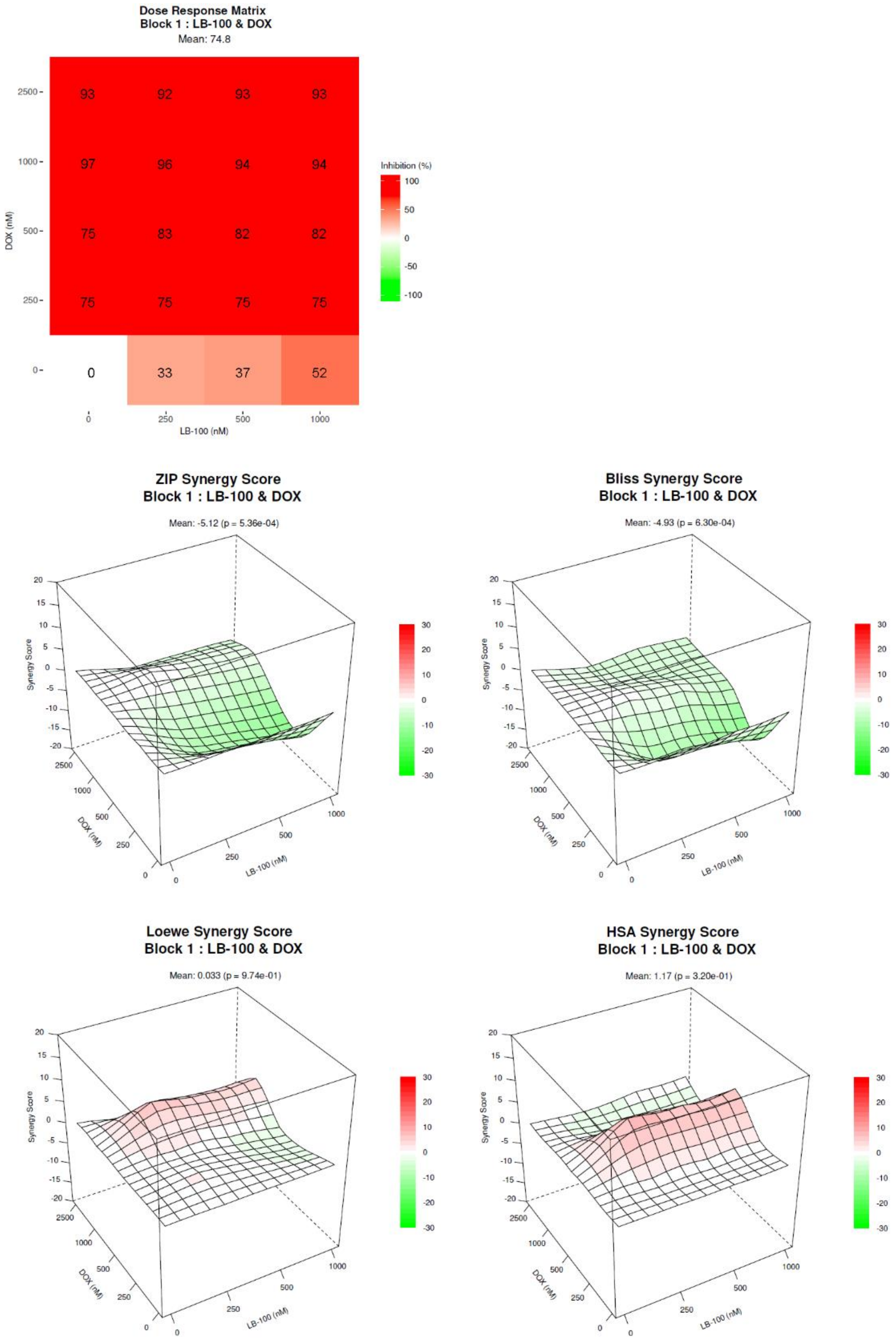

Figure S5. Multiple reference models (ZIP, Bliss, Loewe, and HAS) were generated using SynergyFinder+ based on the Dose Response Matrix derived from the combination treatment of LB-100 and doxorubicin (DOX) in NSCLC patient-derived cells (TR159). Cytotoxic effects were evaluated through an immunofluorescence assay, as detailed in the Materials and Methods section. Positive Synergy Scores, indicated in red, reflect significant synergistic interactions, whereas negative Synergy Scores, shown in green, denote antagonistic effects.

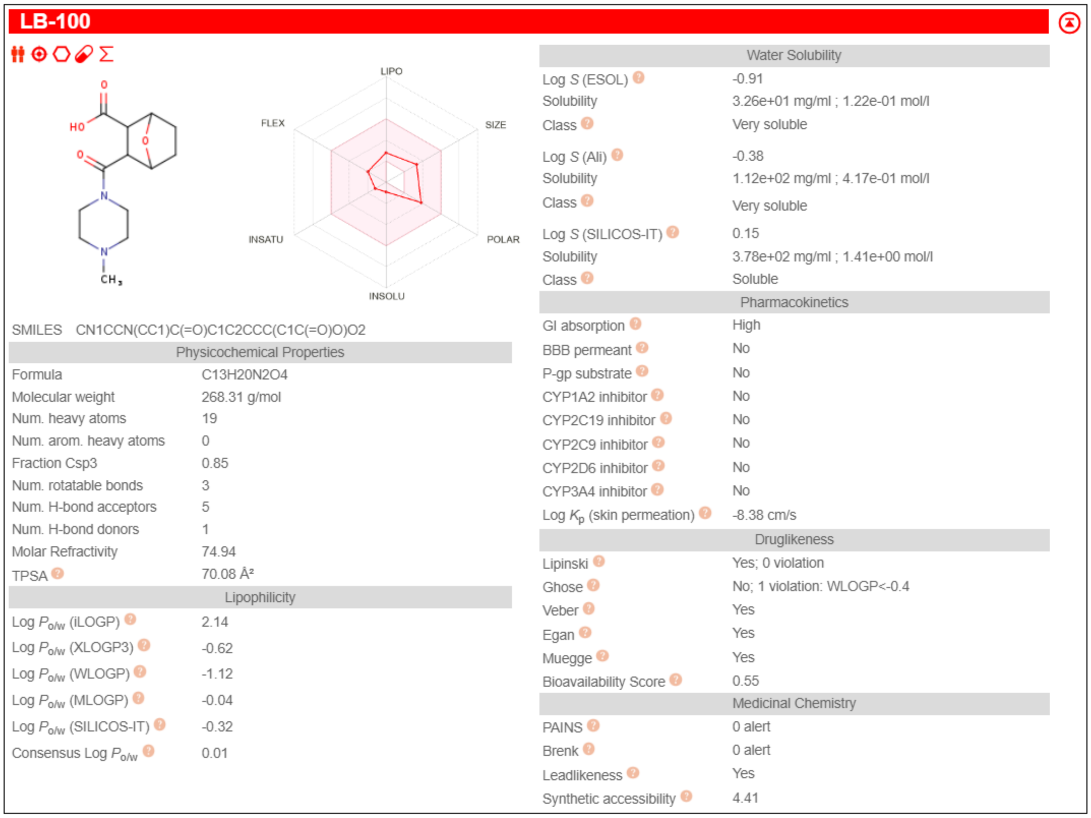

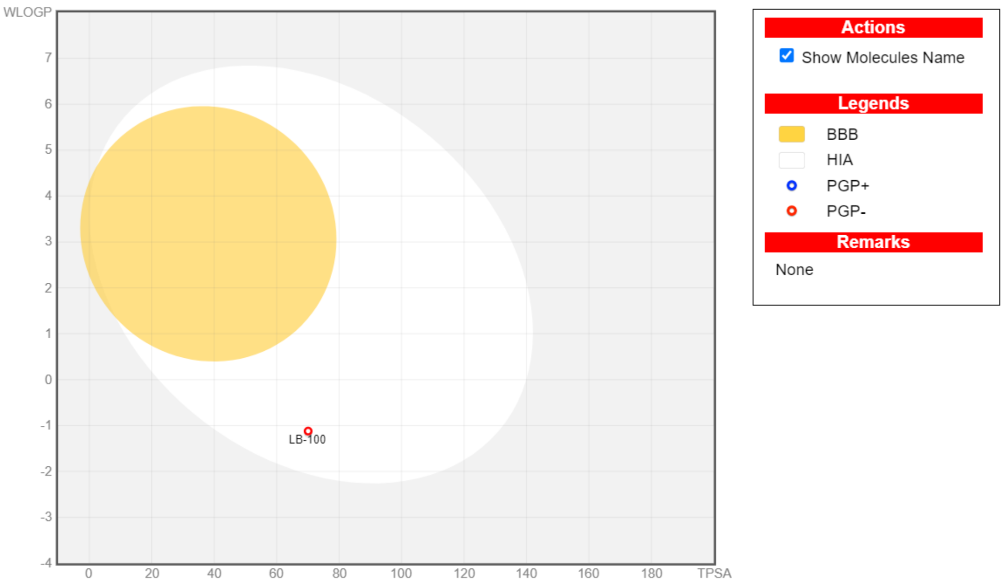

Figure S6. The "boiled-egg" plot—representing the Brain or Intestinal Estimated Permeation Predictive Model—was constructed for LB-100 utilizing the SwissADME online tool. In this representation, the yellow region (analogous to the yolk of a boiled egg) delineates compounds anticipated to undergo passive permeation across the blood-brain barrier (BBB). Conversely, the white region (corresponding to the egg white) signifies molecules projected to be passively absorbed via the gastrointestinal tract (HIA). Blue dots on the plot indicate compounds expected to be P-glycoprotein (P-gp) substrates, while red dots represent those predicted not to interact with P-gp as substrates.

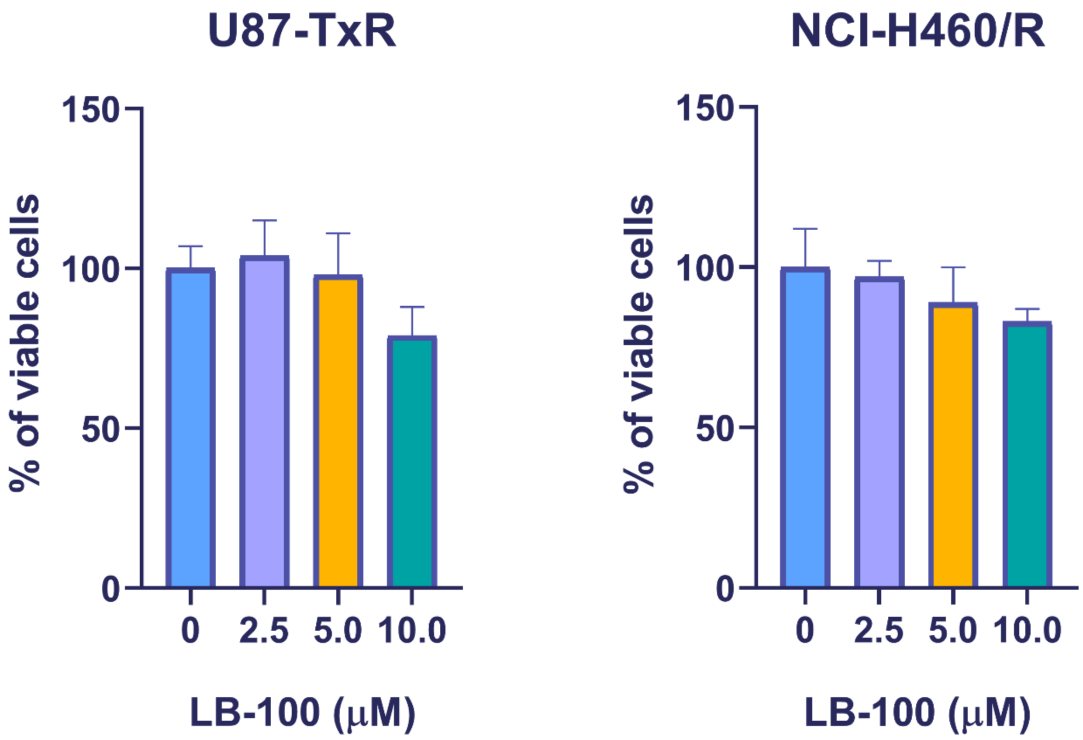

Figure S7. The impact of LB-100 on multidrug-resistant (MDR) cancer cell lines, glioblastoma U87-TxR and non-small cell lung carcinoma NCI-H460/R. Cell viability was evaluated using flow cytometry, employing a forward scatter (FSC) versus side scatter (SSC) plot to delineate the viable cell population from the control sample of untreated cells. The established gating parameters were subsequently applied to the treated cell samples. A minimum of 10,000 events were recorded for each sample to ensure statistical robustness.

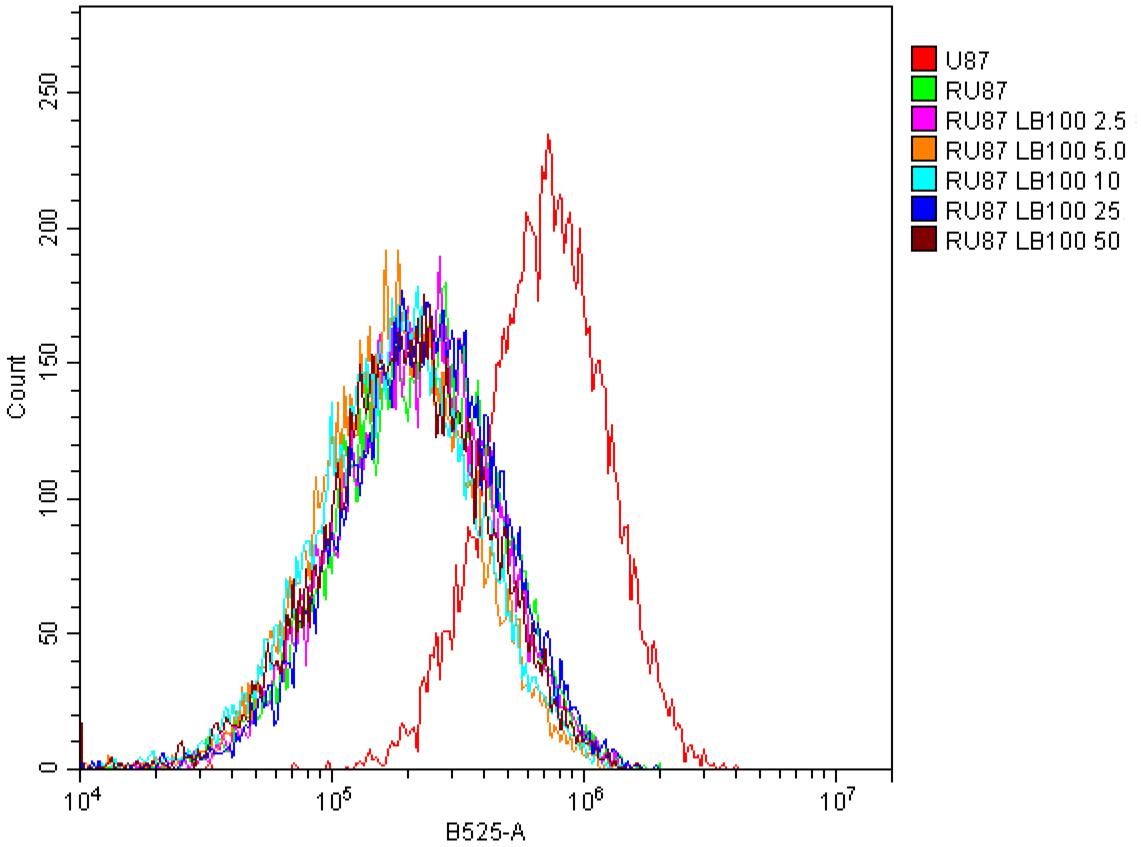

Figure S8. Rhodamine 123 accumulation after LB-100 treatment was evaluated in multidrug-resistant U87-TxR (RU87) glioblastoma cells, which overexpress P-glycoprotein (P-gp). Sensitive U87 cells, which do not express P-gp, served as a positive control for Rhodamine 123 accumulation.

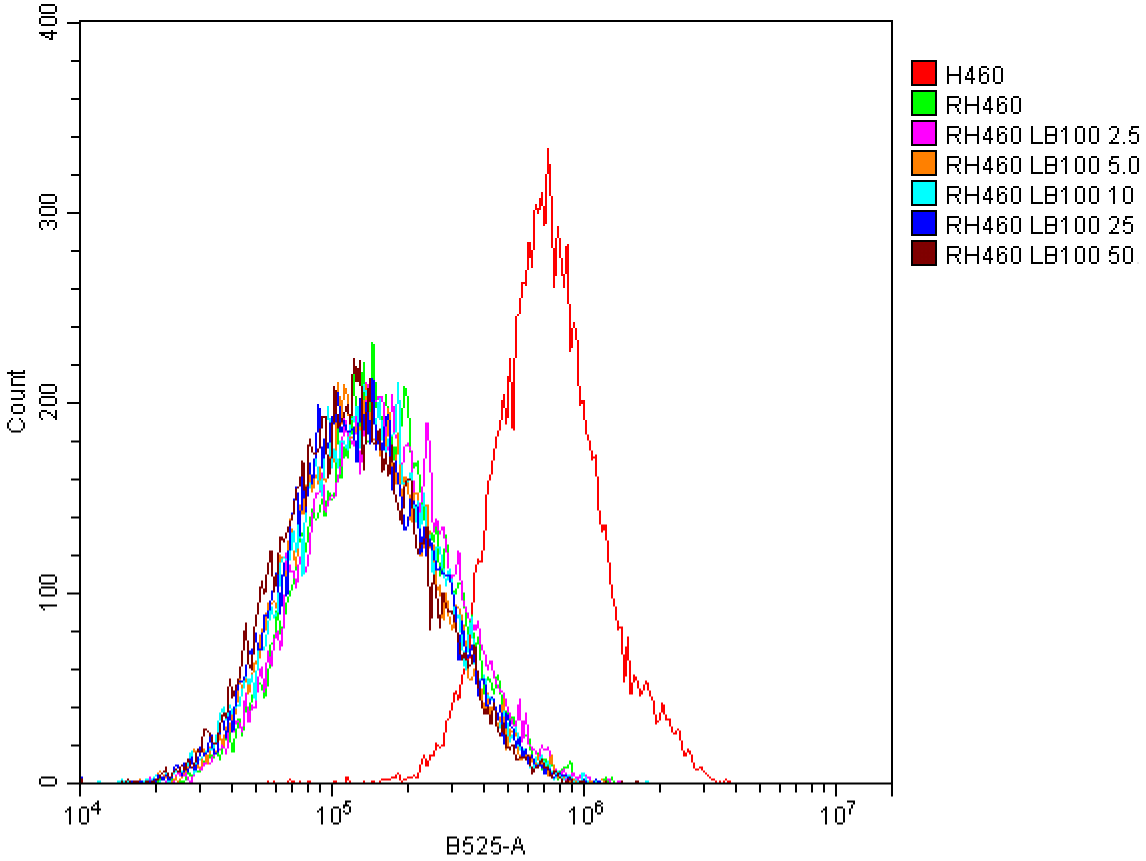

Figure S9. Rhodamine 123 accumulation after LB-100 treatment was evaluated in multidrug-resistant NCI-H460/R (RH460) non-small cell lung carcinoma cells, which overexpress P-glycoprotein (P-gp). Sensitive NCI-H460 (H460) cells, which do not express P-gp, served as a positive control for Rhodamine 123 accumulation.

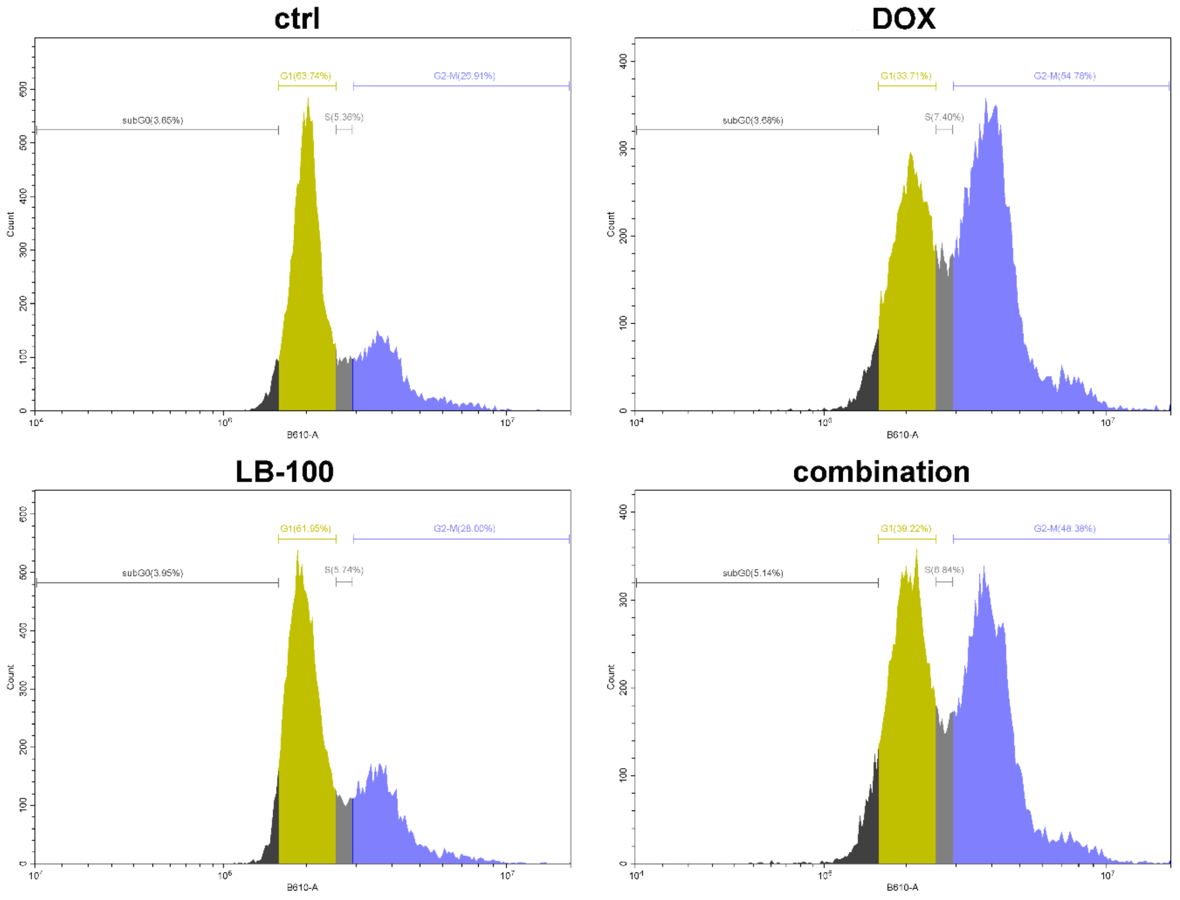

Figure S10. The cell cycle distribution of multidrug-resistant U87-TxR glioblastoma cells was evaluated via propidium iodide (PI) staining following combined treatment with LB-100 and doxorubicin (DOX). The analysis was conducted using flow cytometry, with results interpreted through CytExpert software. In the subsequent treatment regimen, U87-TxR cells were first exposed to 2.5 μM LB-100 for 24 h, after which they were treated with 250 nM DOX, and the duration for this combined treatment extended for an additional 48 h. LB-100 in a single treatment was administered over an entire 72-h period, while the DOX treatment lasted the last 48 h. Representative flow cytometry plots illustrate the outcomes of these treatments.

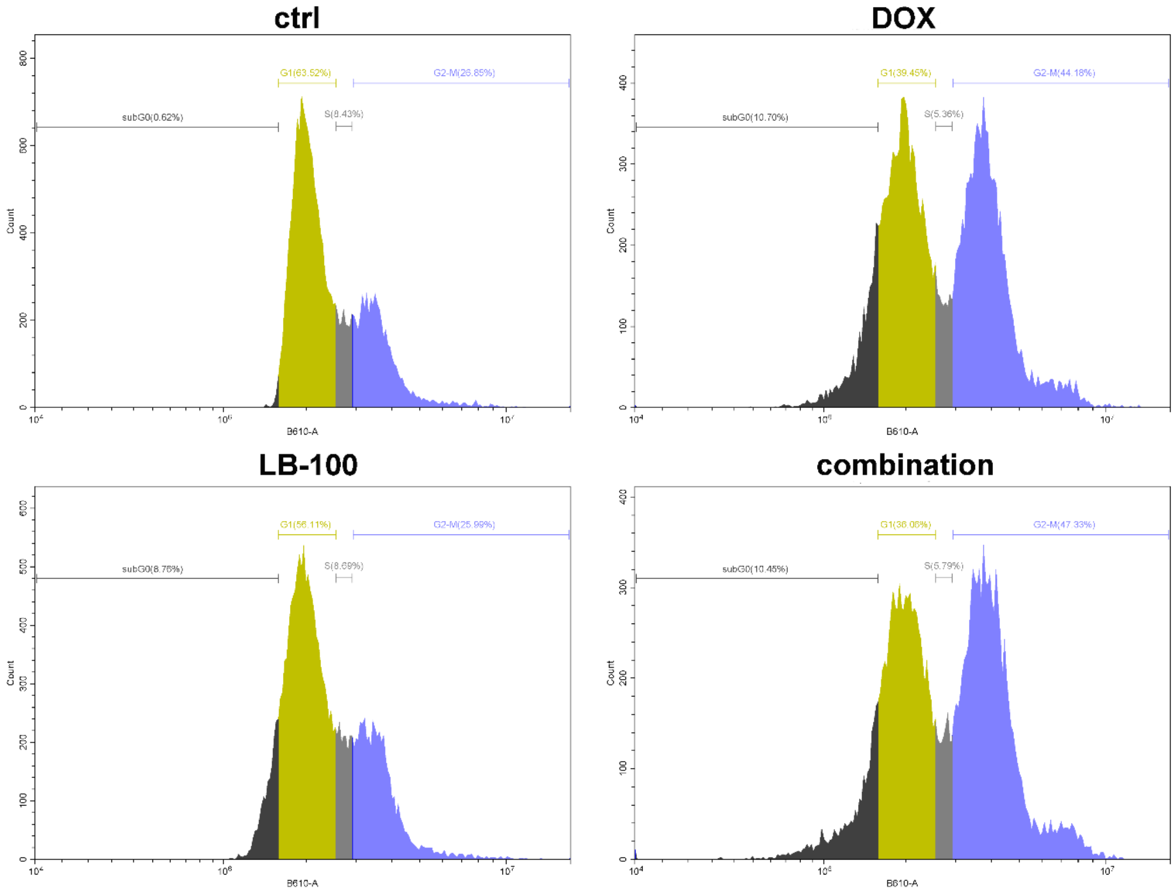

Figure S11. The cell cycle distribution of multidrug-resistant NCI-H460/R non-small cell lung carcinoma cells was evaluated via propidium iodide (PI) staining following combined treatment with LB-100 and doxorubicin (DOX). The analysis was conducted using flow cytometry, with results interpreted through CytExpert software. In the subsequent treatment regimen, NCI-H460/R cells were first exposed to 5.0 μM LB-100 for 24 h, after which they were treated with 500 nM DOX, and the duration for this combined treatment extended for an additional 48 h. LB-100 in a single treatment was administered over an entire 72-h period, while the DOX treatment lasted the last 48 h. Representative flow cytometry plots illustrate the outcomes of these treatments.

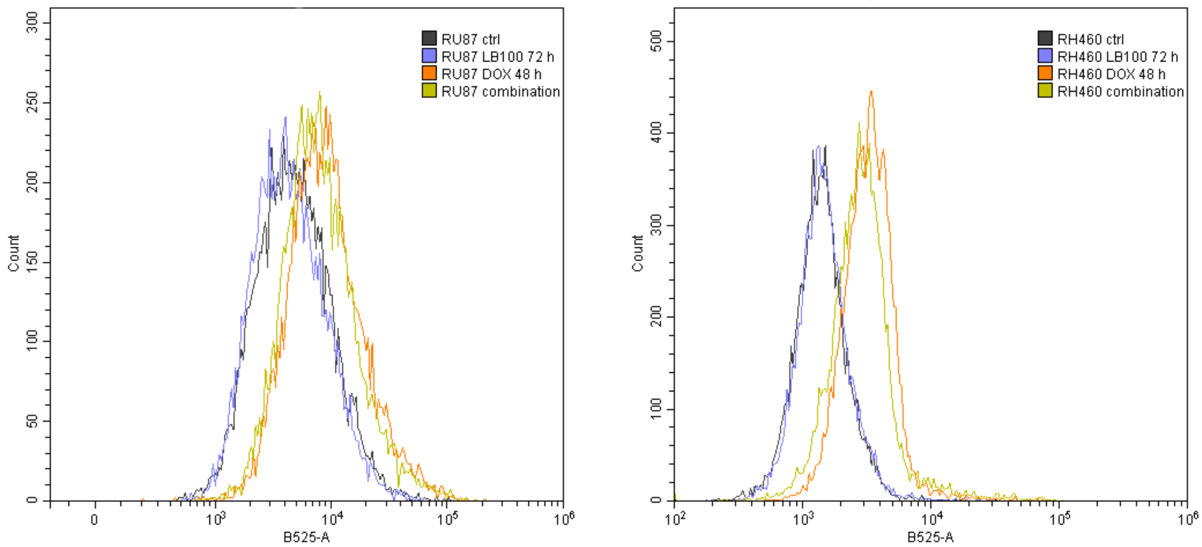

Figure S12. DNA damage was assessed by quantifying the expression levels of phospho-histone 2A.X (pH2A.X) in multidrug-resistant U87-TxR (RU87) glioblastoma and NCI-H460/R non-small cell lung carcinoma cells. In the experimental protocol, U87-TxR and NCI-H460/R cells were initially treated with LB-100 at concentrations of 2.5 μM and 5.0 μM, respectively, for 24 h. Following this exposure, the cells underwent doxorubicin (DOX) treatment at concentrations of 250 nM and 500 nM, respectively, with the combination treatment continuing for an additional 48 h. LB-100 was administered as a single treatment over 72 h, whereas DOX was applied during the final 48 h of this regimen. Subsequently, the cells were labeled using a pH2A.X-specific antibody to evaluate the extent of DNA damage.
